# A Mutation in the FHA Domain of *Coprinus cinereus* Nbs1 Leads to Spo11-Independent Meiotic Recombination and Chromosome Segregation

**DOI:** 10.1534/g3.113.007906

**Published:** 2013-11-01

**Authors:** K. Nicole Crown, Oleksandr P. Savytskyy, Shehre-Banoo Malik, John Logsdon, R. Scott Williams, John A. Tainer, Miriam E. Zolan

**Affiliations:** * Department of Biology, Indiana University, Bloomington, Indiana 47405; † Department of Biology, University of Iowa, Iowa City, Iowa 52242; ‡ National Institute of Environmental Health Sciences, Research Triangle Park, North Carolina 27709; § The Scripps Research Institute, La Jolla, California 92037

**Keywords:** MRN complex, *Coprinus cinereus*, recombination, DNA replication, meiosis

## Abstract

Nbs1, a core component of the Mre11-Rad50-Nbs1 complex, plays an essential role in the cellular response to DNA double-strand breaks (DSBs) and poorly understood roles in meiosis. We used the basidiomycete *Coprinus cinereus* to examine the meiotic roles of Nbs1. We identified the *C. cinereus nbs1* gene and demonstrated that it corresponds to a complementation group previously known as *rad3*. One allele, *nbs1-2*, harbors a point mutation in the Nbs1 FHA domain and has a mild spore viability defect, increased frequency of meiosis I nondisjunction, and an altered crossover distribution. The *nbs1-2* strain enters meiosis with increased levels of phosphorylated H2AX, which we hypothesize represent unrepaired DSBs formed during premeiotic replication. In *nbs1-2*, there is no apparent induction of Spo11-dependent DSBs during prophase. We propose that replication-dependent DSBs, resulting from defective replication fork protection and processing by the Mre11-Rad50-Nbs1 complex, are competent to form meiotic crossovers in *C. cinereus*, and that these crossovers lead to high levels of faithful chromosome segregation. In addition, although crossover distribution is altered in *nbs1-2*, the majority of crossovers were found in subtelomeric regions, as in wild-type. Therefore, the location of crossovers in *C. cinereus* is maintained when DSBs are induced via a Spo11-independent mechanism.

The faithful propagation of genetic material from one generation to the next depends on mechanisms that accurately replicate and package chromosomes into a new cell. Production of haploid gametes from diploid cells requires special consideration because each gamete must receive only one copy of the genome. To solve this problem, cells undergo a specialized cell division called meiosis in which replication is followed by two rounds of cell division. During meiosis I, homologous chromosomes pair and undergo recombination. Recombination is initiated by the formation of programmed DNA double-strand breaks (DSBs), made by the conserved meiosis-specific protein Spo11, which are repaired by an interaction with a homologous chromosome (Keeney *et al.* 1997; Keeney 2001; Malik *et al.* 2007). In both *Schizzosaccharomyces pombe* and *Saccharomyces cerevisiae*, the 5′ ends of the DSB are resected into single-strand ends by the combined activities of the Mre11-Rad50-Nbs1 (MRN) complex, Ctp1 (called Sae2 in *S. cerevisiae*) and other nucleases (Mimitou and Symington 2009; Nicolette *et al.* 2010). The new single-strand end invades the homolog and DNA synthesis begins using the homolog as the template (Neale and Keeney 2006). Strand invasion and synthesis displace one strand of the DNA duplex, termed the D-loop, and the noninvading strand of the DSB pairs with the D-loop and primes another round of synthesis. The end product is a joint molecule, which in *S. cerevisiae* is a double Holliday junction (Schwacha and Kleckner 1995) and in *S. pombe* is a single Holliday junction (Cromie *et al.* 2006). The Holliday junctions (or junction) are resolved into a crossover, which is the reciprocal exchange of genetic material flanking the Holliday junctions. Crossovers are then stabilized into chiasmata by sister-chromatid cohesion.

Crossover control, which ensures that each pair of homologs receives a crossover, is a highly regulated process. The number of crossovers on a chromosome and the location of crossovers are both critical factors that contribute to successful homolog segregation. In most organisms, every pair of homologs must receive at least one crossover for proper disjunction during anaphase I, but chromosomes frequently have more than one crossover. When there are multiple crossovers per chromosome, they are distributed nonrandomly along the chromosome. This is referred to as crossover interference, whereby a crossover in one genetic interval decreases the likelihood of a crossover occurring in the neighboring genetic interval (see Berchowitz and Copenhaver 2010 for a full discussion of crossover interference). Crossover homeostasis ensures that the number of crossovers in the genome remains constant independently of the number of DSBs that are made (Hillers and Villeneuve 2003; Martini *et al.* 2006; Cole *et al.* 2012). Whether crossover homeostasis and crossover interference are manifestations of a single crossover control mechanism or of distinct processes is undergoing debate (Cole *et al.* 2012).

The MRN complex is a central mediator in all types of DSB repair, because it is necessary for meiotic recombination, nonhomologous end joining, microhomology-mediated end joining, V(D)J recombination, telomere maintenance, repair of DSBs incurred during replication fork collapse, and homologous recombination (Moore and Haber 1996; Tsukamoto *et al.* 1996; Costanzo *et al.* 2001; Williams *et al.* 2010). Mre11 is a homolog of bacterial SbcD (Sharples and Leach 1995) and has 3′→5′ exonuclease and endonuclease activities (D’Amours and Jackson 2002 and references therein). In *S. cerevisiae*, during meiotic recombination, the endonucleolytic activity of Mre11 is required to nick the DNA, and the exonucleolytic activity is required to resect the DNA toward the Spo11-bound DNA end (Takeda *et al.* 2007; Garcia *et al.* 2011). Rad50 has ATPase activity and hinged coiled–coiled regions, and acts to tether the ends of the DSB together (Alani *et al.* 1989; De Jager *et al.* 2001; Hopfner *et al.* 2002). The third member of the complex, Nbs1, has several functions. In mammalian cells, it brings Mre11 and Rad50 into the nucleus and localizes the complex to sites of damage (Tauchi *et al.* 2001; Chapman and Jackson 2008; Melander *et al.* 2008; Spycher *et al.* 2008). In both mammalian cells and *S. cerevisiae*, Nbs1 (Xrs2 in *S. cerevisiae*) has been shown to act as the signaling component of the complex and communicates the presence of a break to the cell by activating ATM, the central mediator in the DNA damage response (Usui *et al.* 2001; Lee and Paull 2004; Lee and Paull 2005). Nbs1 recruitment of ATM can influence Mre11-dependent DNA end-degradation and microhomology-mediated end joining for repair in human cells (Rahal *et al.* 2010). In *S. pombe*, Nbs1 also directly binds and recruits Ctp1 to DSBs (Lloyd *et al.* 2009; Williams *et al.* 2009).

Although Nbs1 is conserved among animals, plants, and fungi, the protein is rapidly diverging (Demogines *et al.* 2010). Despite this, several domains are present in all homologs. The N-terminus contains a Forkhead-associated (FHA) domain and two tandem BRCA C-terminus (BRCT) domains, whereas the C-terminus contains the Mre11-binding motif and an ATM-binding motif (Lloyd *et al.* 2009; Williams *et al.* 2009). FHA and BRCT domains mediate phosphorylation-dependent protein–protein interactions and are frequently found in DNA repair proteins (Durocher and Jackson 2002; Glover *et al.* 2004). In mammalian cells, the FHA domain of Nbs1 is required for irradiation-induced focus formation, and together the FHA and BRCT domains bind MDC1, facilitating localization and retention of the MRN complex to sites of damaged chromatin (Tauchi *et al.* 2001; Chapman and Jackson 2008; Melander *et al.* 2008; Spycher *et al.* 2008; Wu *et al.* 2008). There are no identified fungal orthologs of Mdc1, so it remains unknown with which proteins, if any, Nbs1 interacts to recruit the MRN complex to a break in these organisms. In addition to binding Mdc1, the FHA domain of Nbs1 contains a binding pocket, which in *S. pombe* is known to interact with phosphorylated residues on Ctp1 to recruit it to the break (Lloyd *et al.* 2009; Williams *et al.* 2009). The C-terminus of Nbs1 contains the Mre11 and ATM binding motifs, but has otherwise been shown to be disordered and lacking any globular domains (Lloyd *et al.* 2009; Williams *et al.* 2009; Schiller *et al.* 2012).

Although the DNA repair roles of Nbs1 have been well-characterized, less is known about its roles in meiosis. In *S. cerevisiae*, Mre11, Rad50, and Xrs2 are required for both meiotic DSB formation and processing (Symington 2002). In other organisms, meiotic DSB formation is MRN-independent, but DSB processing requires the activities of MRN (Riha *et al.* 2004; Smith *et al.* 2004; Mehrotra and McKim 2006; Hayashi *et al.* 2007; Acharya *et al.* 2008; Waterworth *et al.* 2007). In yeast, mouse, and *Drosophila melanogaster*, Nbs1 activation of ATM has been shown to inhibit Spo11 activity, thus providing a negative feedback loop to control DSB formation (Carballo *et al.* 2013; Joyce and McKim 2011; Lange *et al.* 2011). Xrs2, in *S. cerevisiae*, serves as a scaffold to recruit Pch2, a protein that has many meiotic functions, including axis morphogenesis and checkpoint control (Ho and Burgess 2011). More generally, mutations in *nbs1* lead to spore viability defects in fungi (Semighini *et al.* 2003; Shima *et al.* 2005) and viable mouse mutants show mild chromosome synapsis defects (Cherry *et al.* 2007). Mutations in Ctp1 that prevent interaction with the FHA domain of *S. pombe* Nbs1 cause a spore viability defect as well (Dodson *et al.* 2010).

*Coprinus cinereus (Coprinopsis cinerea*) (Redhead *et al.* 2001) is a basidiomycete that has a naturally synchronous meiosis, sophisticated cytogenetics, and a fully annotated genome, making it a powerful experimental system for the study of meiosis. *C. cinereus mre11* and *rad50* were initially identified in UV mutagenesis screens as members of an epistasis group that was radiation-sensitive and defective in meiosis (Zolan *et al.* 1988; Valentine *et al.* 1995; Zolan *et al.* 1995; Gerecke and Zolan 2000; Acharya *et al.* 2008). One of the genes in the epistasis group, *rad3*, was never identified (Zolan *et al.* 1988). The *rad3* complementation group consists of five alleles. Four of these alleles, *rad3-1*, *rad3-3*, *rad3-4*, and *rad3-5*, fail to make viable spores, are severely radiation-sensitive, and have defects in chromosome condensation, axial element formation, and synaptonemal complex formation (Zolan *et al.* 1988; Pukkila *et al.* 1992; Valentine *et al.* 1995; Zolan *et al.* 1995). In contrast, *rad3-2* was shown to be only mildly radiation-sensitive, and it has a spore viability of 58%. It displayed a hyper-recombination phenotype in one genetic interval and a mild chromosome condensation defect, and it made apparently normal synaptonemal complex (Zolan *et al.* 1988). In this work, we identify *C. cinereus rad3* as *nbs1* and show the effects of a mutation predicted to be in the FHA domain at a site critical for interaction with other proteins. This mutation causes a change in the meiotic crossover frequency and distribution. Surprisingly, crossovers are apparently Spo11-independent in this mutant, and instead likely result from unrepaired DSBs made during premeiotic replication. Our studies demonstrate remarkable flexibility in the meiotic process and point to a role for the Nbs1 FHA domain as part of the Mre11-Rad50-Nbs1 complex in modulating replication fork processing and protection and DSB repair.

## Materials and Methods

### Strains and culture conditions

The wild-type strains of *C. cinereus* used in this study were *J6;5-4* and *J6;5-5* (Valentine *et al.* 1995). The following *rad3* strains were used: *rad3-1;5-7, rad3-1;5-15, rad3-2;5-3, rad3-2;5-7, rad3-2;5-8, rad3-3;5-5, rad3-3;5-15, rad3-5;5-30,* and *rad3-5;5-44* (Zolan *et al.* 1988; Valentine *et al.* 1995). The *rad3-2* strains used for genotyping were created by mating *rad3-2;5-3* to *HT 14.01 #172 A6B6 trp1* (Stajich *et al.* 2010) and isolating a spore that had the *rad3-2* mutation but a mostly *172* genetic background. This new strain, which we call *rad3-2 #63,* was mated to *rad3-2;5-7* to form the homokaryon mushroom used to generate spores suitable for genetic mapping. The *rad3-2 #63* was also crossed to *rad3-2;5-8* to generate a heterokaryon mushroom for genetic mapping; *rad3-2;5-8* is a wild-type sibling strain isolated from the crosses that generated the other *rad3-2* strains. The wild-type cross used for genetic mapping was *J6;5-4 × HT 14.01 #172. spo11-1 #63* and *spo11-1 #74* were used for microscopy (Celerin *et al.* 2000). To generate the *spo11-1;nbs1-2* double mutant, *spo11-1 #74* was crossed to *nbs1-2;5-2*. Culture conditions, mating, and fruiting conditions are as described by Zolan *et al.* (1988).

### Identifying *nbs1* in *C. cinereus*

*C. cinereus nbs1* was identified by consecutive tBLASTn and PSI-BLASTp (Altschul *et al.* 1997) searches of the *C. cinereus* genome database (http://genome.semo.edu/ccin). Initial tBLASTn searches with *Ustilago*, *Podospora*, *Neurospora*, and *Gibberella* homologs (Genbank accession numbers EAK82013.1, CAD60586.1, XP_325904.1, and EAA76696.1) were performed because queries revealed divergent homologous *C. cinereus* predicted genes retrain_ccin_Contig226-snap.8 and retrain_ccin_Contig226-snap.7 (these predicted genes are named according to the nomenclature used in the *C. cinereus* GBrowse). Next, predicted amino acid sequences of 25 fungal Nbs1 orthologs identified by PSI-BLASTp from Genbank were aligned by ClustalX (Chenna *et al.* 2003), and this alignment was used for a more thorough and sensitive search of the *C. cinereus* genome by PSI-BLASTp using a custom position-specific scoring matrix (PSSM). This search identified the N-terminal 300 amino acids of *C. cinereus* Nbs1, corresponding to the predicted gene CC1G_12337 (XP_002910082.1) in the *C. cinereus* GBrowse site (http://genome.semo.edu/ccin). We identified two errors in intron splicing sites of the predicted *nbs1* sequence by sequencing cDNA and have deposited the correct sequence in NCBI (Genbank accession number JQ917485).

To verify that *C. cinereus* Nbs1 groups with other fungal homologs, we performed phylogenetic analyses. PSI-BLASTp was used to identify homologous Nbs1 amino acid sequences from all diverse eukaryotes at the Genbank nonredundant database. Their multiple sequence alignment was constructed using MUSCLE version 3.7 (Edgar *et al.* 2004) and adjusted and edited manually in MacClade version 4.08 (Maddison *et al.* 2006), only using the 186 unambiguously aligned amino acid sites for phylogenetic analyses, with constant sites removed. Phylogenetic trees were estimated using RAxML version 7.3.1 (Stamatakis *et al.* 2006; Stamatakis *et al.* 2008), hosted by the CIPRES Science Gateway Portal version 3.1 at the San Diego Supercomputer Center (Miller *et al.* 2010) (http://www.phylo.org/portal/). The LG model for amino acid substitutions (Le *et al.* 2008) was used, and among-site substitution rate heterogeneity was corrected using 25 γ-distributed substitution rate categories (LG+25γ). Support was inferred from 1000 bootstrap replicates.

### RNA extraction and reverse-transcription PCR

To confirm the *nbs1* gene structure and sequence as predicted by the Broad Institute genome sequence, reverse-transcription (RT) PCR was used on purified poly-A RNA from a J6;5x4 K+6 mushroom (SuperScript One-Step RT-PCR for Long Templates, Invitrogen; PolyATtract mRNA Isolation System III, Promega). Primers are listed in Supporting Information, Table S1. RT-PCR conditions were as follows. The reaction mixture contained 20 µl water, 25 µl 2× reaction mix, 1 µl of each primer (100 µM stock), 2 µl RNA (5–10 ng/µl), and 1 µl RT-Taq. Thermocycler conditions were 50° for 30 min, 94° for 2 min, then 40 cycles of 94° for 30 sec, 55° for 30 sec, and 68° for 3.5 min, then a final extension at 72° for 7 min, and a 4° hold. RT-PCR products were ligated into pCR2.1 vector and transformed into Top-10 *E. coli* cells (Topo-TA cloning kit, Invitrogen). Individual colonies were picked, grown in 5 ml LB media overnight, and then the plasmid was extracted using the Qiaprep Spin Miniprep Kit (Qiagen). The plasmid containing the cDNA of *nbs1* was then sequenced using primers from Table 1. Sequencing conditions were as follows. The reaction mixture contained 4 µl water, 4 µl 5-mM magnesium chloride, 1 µl 100-uM primer, 1 µl plasmid DNA, and 1 µl BigDye sequencing mix (Applied Biosystems). Completed sequencing reactions were given to the Indiana Molecular Biology Institute to be analyzed on the ABI 3730 (Applied Biosystems). Sequences were analyzed using CodonCode Aligner version 3.7.1 (CodonCode Corporation).

**Table 1:** Mutations in *nbs1* alleles

Allele	Mutation	Location (bp)	Effect on Protein
*nbs1-1*	A→G	1235	In intron splice site, early truncation after amino acid 400
*nbs1-2*	CC→TT	188–189	Serine → phenylalanine at amino acid 43
*nbs1-3*	C→T	2126	Early truncation after amino acid 570
*nbs1-4*	C→T	2126	Early truncation after amino acid 570
*nbs1-5*	GGCACT→GGTA-T	1072–1074	Early truncation after amino acid 288

### Amplifying *nbs1* from genomic DNA

*nbs1* from genomic DNA of Java 6 and strains of the *rad3* complementation groups were amplified using the primers from Table S1. PCR conditions were as follows. The reaction mixture contained 34 µl water, 10 µl 5× reaction buffer, 2.5 µl 2.5-mM stock solutions of each dNTP, 1 µl of each primer (100 µM), 1 µl DNA (30–500 ng/µl, depending on reaction), and 0.5 µl Taq (Roche Expand High Fidelity Plus PCR System). Thermocycler conditions were 90° for 5 min, then 35 cycles of 95° for 30 sec, 55° for 30 sec, and 72° for 30 sec per kilobase of target fragment, then a final extension at 72° for 10 min, followed by a hold at 10°. PCR products were resolved on a 1% agarose gel stained with ethidium bromide and then purified using either a gel purification kit (Qiagen Qiaquick Gel Extraction Kit) or a PCR clean-up kit (Zymo Research DNA Clean and Concentrate-5). Clean PCR products were sequenced using the same conditions previously mentioned.

### Complementation of *rad3-1* with *nbs1*

A full-length *nbs1* construct containing 1000 bp upstream and 450 bp downstream of *nbs1* was amplified from Java 6 genomic DNA. A touchdown PCR was used starting at 63° and ending at 50° for a total of 45 cycles using primers listed in Table S1. The PCR product was cloned into pCR2.1 using Topo-TA cloning (Invitrogen).

The *nbs1* construct was co-transformed with pCC1001 (a *trp1*-containing vector) (May *et al.* 1991) into a *rad3-1*; *trp1-1,1-6* (Binninger *et al.* 1987) strain as follows. The *rad3-1*; *trp1-1,1-6* was grown on YMG plates for 10 d until confluent. Oidia from three 100-mm × 15-mm plates were collected in 10 ml sterile water by gentle agitation with a glass pipette and then filtered through sterile glass wool into a Falcon tube. Oidia were collected in a pellet by centrifugation in an IEC centrifuge at 2000*g* for 5 min and resuspended in 5 ml MM buffer (1 M mannitol, 0.2 M maleic acid, pH 5.5). Oidia were collected by centrifugation again and resuspended in 2 ml per plate harvested of Vinoflow (60 mg/ml Vinoflow, Novozymes). Oidia were allowed to protoplast for approximately 1 hr at 37° with gentle shaking every 20 min. Protoplasts were filtered through glass wool, collected by centrifugation for 5 min, and resuspended in 2 ml MMC (1 M mannitol, 0.2 M maleate, 0.1 M CaCl_2_) per plate harvested; 100 µl protoplasts were mixed with 25 µl PEG solution (2.5% PEG 4000, 10 mM Tris pH 7.5, 0.1 M CaCl_2_) and either 1 µg each of the *nbs1* construct and pCC1001 or pCC1001 only as a positive control, and then incubated on ice for 30 min. An additional 1 ml PEG solution was added and incubated for 5 min at room temperature, then 2 mL MMC was added and mixed gently. Protoplasts were divided evenly onto six plates of regeneration agar and placed at 37°. Transformants were picked as they appeared over the next few days.

A radiation sensitivity screen was performed by subculturing transformants onto YMG plates, exposing them to 40 krad and 60 krad of ionizing radiation (^137^Cs source, model Mark I-68A, J.L. Shephard and Associates) and screening 2 d later for regrowth after exposure.

### Spore viability

Mushrooms were allowed to fruit and the spores from a single mushroom were collected in sterile water and filtered through glass wool to remove any cap tissue. The spores were centrifuged for 5 min at 2000*g*, resuspended in 2 ml water, and then serially diluted by a factor of 10. Five 1-µl drops of each dilution were spotted onto YMG plates that contained 0.01% furfural and allowed to germinate overnight for 16 hr. The number of spores that germinated per 1-µl spot was counted by eye using a dissecting microscope and this number was divided by the total number of spores in the spot. In general, the 10^−2^ dilution provided approximately 50 spores per spot, and this dilution was used for counting. The spore viability was calculated by averaging the percent germination for each of the five spots.

### Tetrad analysis

Mushrooms were allowed to fruit and a gill peel was taken 24 hr after karyogamy. The gill was laid flat on a sterile glass slide and allowed to dry for at least 1 hr. To isolate tetrads, the slide was glued to an empty Petri dish and turned upside-down on a micromanipulator. A single tetrad was picked and moved to a 1-cm × 1-cm × 0.5-cm slab of YMG containing 0.01% furfural. Once on the slab of YMG, the individual spores of the tetrad were separated using a micromanipulator. This slab of YMG was then transferred to another YMG plate to keep the slab moist. Spores were allowed to germinate overnight in a humid chamber at 37°.

### Generation of polymorphic strains used for genetic mapping

Strains used for genotyping are as described in the *Strains and culture conditions* section. One hundred spores from the *nbs1-2* and wild-type crosses were isolated and used for genetic mapping. The total sample size for any genetic interval varies and is slightly less than 100, because PCR reactions for some samples would occasionally fail. Importantly, for intervals tssr74/tssr77 and tssr93/tssr98 on chromosome 3 and for all intervals examined on chromosome 8, data from two separate wild-type mushrooms were combined. The map length and crossover distribution were not different between the two mushrooms, so the data from the two were combined into one dataset (Fisher exact tests, all intervals *P* > 0.1; chi-square test for all intervals combined on chromosome 8, *P* = 0.67).

### DNA extractions

DNA extractions were performed as described by Walser *et al.* (2002).

### Genotyping

To determine the recombination rates on chromosomes 3 and 8, we used genetic mapping of simple sequence repeats (SSRs), single nucleotide polymorphisms (SNPs), and insertion deletion polymorphisms. We determined the length of the simple sequence repeats in the parental strains used to generate the crosses (Table S3) and then determined the lengths of the SSRs in their offspring. For SNPs and insertion deletion polymorphisms, we determined the genotypes of the parental strains and their offspring. A crossover event was determined by noting the intervals in which the genotype of an offspring switched from that of one parent to that of the other.

Each of the polymorphic SSRs was amplified using a nested PCR approach (Schuelke 2000). The forward primer for each SSR was designed with an M13(-21) tail on the 5′ end (Table S2). In the same PCR reaction, an M13(-21) universal primer with the fluorescent dye VIC (Applied Biosystems) attached was added. This enabled the economic amplification of multiple SSRs using the same fluorescently labeled primer. PCR reactions contained 30 ng DNA, 4.7 µl water, 2 µl GoTaq 5× buffer, 1.6 µl dNTPs (2.5 mM), 0.2 µl M13-VIC universal primer (10 µM), 0.2 µl gene-specific forward primer (1 µM), 0.2 µl gene-specific reverse primer (10 µM), and 0.1 µl GoTaq Flexi Polymerase (Promega). DNA from isolated spores was placed in a 96-well plate and one SSR per plate of DNA samples was amplified using the following PCR program. Cycle 1: (1×) 95°, 3 min; cycle 2: (10×) 94°, 30 sec; 58°, 30 sec, decreasing temperature by 1° after every cycle; 72°, 45 sec; cycle 3: (29×) 94°, 30 sec; 48°, 30 sec; 72°, 45 sec; and cycle 4: (1×) 72°, 20 minutes; 10° hold.

The PCR products were diluted 1:200 in water, and then 2 µl diluted PCR was added to 9 µl GeneScan 500 LIZ size standard (Applied Biosystems) that was diluted 1:180. This mixture was then denatured at 95° for 5 min and immediately placed on ice to prevent the PCR products from re-annealing. The sizes of the PCR fragments were determined by electrophoresis and laser detection on an ABI 3730. The software GeneMapper (version 3.7, Applied Biosystems) was used to analyze the fragments and determine which parental allele each progeny had. A peak was accepted if the height was more than 500 units of fluorescence intensity. If there were multiple peaks or the peak was less than 500 units, then the sample was rejected.

### SNP mapping

To locate SNPs, 1000-bp fragments of intergenic regions in the genetic intervals of interest were amplified from the parental generation of the wild-type and mutant crosses (primers are listed in Table S4). These fragments were then sequenced and analyzed for polymorphisms between strains. In the case that an insertion deletion polymorphism was identified, primers were designed to amplify a fragment suitable for genotyping. A complete list of parental SNP alleles is presented in Table S5.

### Statistical analysis

To test for significant differences in the genetic map lengths of individual intervals between wild-type and *nbs1-2*, a Fisher exact test was used from GraphPad QuickCalcs (http://www.graphpad.com/quickcalcs/contingency1.cfm). To assess whether the change in genetic map length of the whole chromosome was statistically different between strains, the chromosome was treated as one interval and a chi-square test was used to test for significant differences. To determine if the distribution of crossovers across an entire chromosome was different between wild-type and *nbs1-2*, a chi-square test of independence with a Monte Carlo simulation (2000 samplings) was used, using the program R (R Development Core Team 2011). Statistical analysis of the anti-gamma-H2AX time course was performed using a two-way ANOVA with Tukey multiple comparison test (GraphPad Prism version 6.00 for Windows, GraphPad Software, La Jolla, CA; www.graphpad.com).

### Antibodies

The anti-gamma-H2AX antibody is commercially available from BioLegend (clone 2F3, catalog number 613402) and was used at 5 µg/ml. A donkey anti-mouse secondary antibody conjugated to DyLight-549 was used at 0.3 µg/ml (catalog number 715-505-150, Jackson ImmunoResearch).

### Chromosome spreads and immunofluorescence

Chromosome spreads and immunofluorescence were performed as described by Acharya *et al.* (2008). Images were analyzed using Metamorph (Molecular Devices) software as follows. To count the number of foci in a given nucleus, the background of the FITC or TRITC channel image was flattened using the “flatten background” function. The background of this new image was subtracted using the “use region for background” function. To avoid counting foci that were background noise from the camera, an inclusion threshold was set by the user that avoided background noise. The number of foci was counted using the “integrated morphometry analysis” tool. For anti-gamma-H2AX staining, foci in the nucleolus were counted and included in the analysis.

### Camptothecin sensitivity

We tested for sensitivity to camptothecin, a DNA-damaging agent that causes DSBs at replication forks by preventing removal of topoisomerase I. For 3 d, *J6;5-4*, *nbs1-2;5-3*, *nbs1-2;5-7*, and *nbs1-1;5-15* were grown on YMG and then subcultured onto minimal medium, minimal medium containing 1 μM camptothecin (Sigma-Aldridge), or minimal medium containing a mock treatment of camptothecin. Camptothecin was first dissolved in DMSO to a concentration of 100 μM, then diluted to 10 μM in 10% ethanol/2% Tween, and added to minimal media at a final concentration of 1 μM. Mock treatment consisted of adding the same volume of DMSO and 10% ethanol/2% Tween to minimal media as was used for camptothecin. Cultures were scored for growth 2 d after subculturing.

## Results

### Identification of *C. cinereus* Nbs1

**Figure 1:**
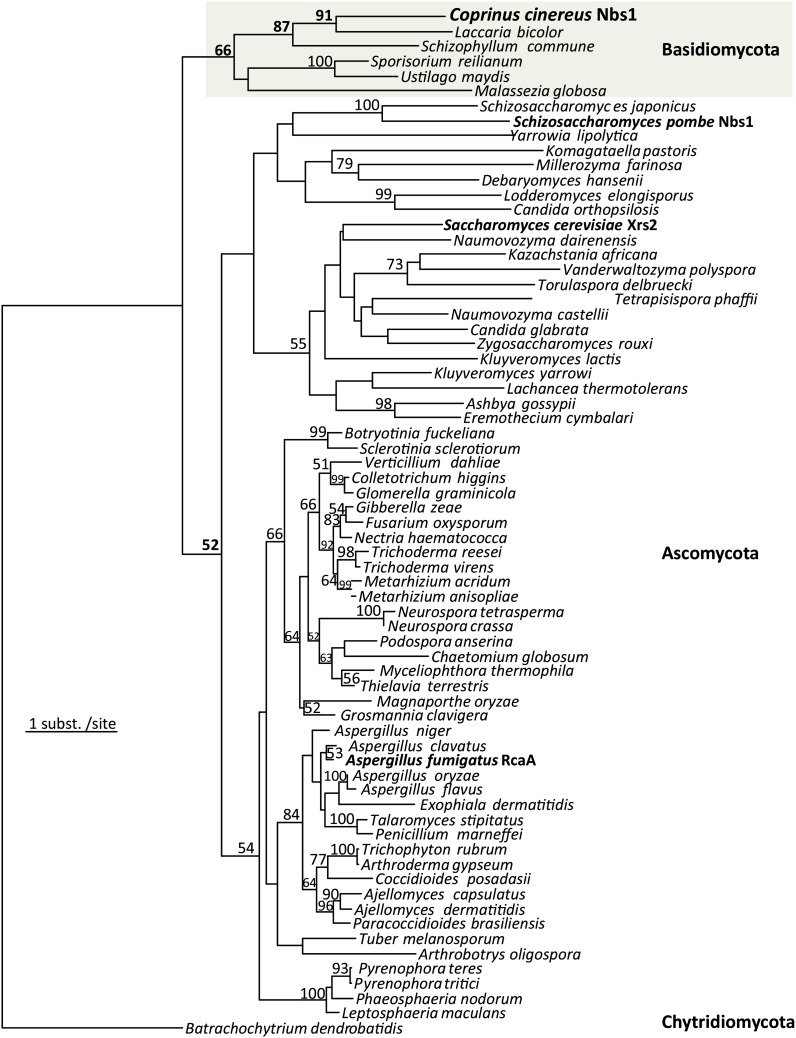
Phylogenetic tree showing the evolutionary relationships among fungal orthologs of *nbs1*. *C. cinereus* Nbs1 groups with its closest relative *Laccaria* and with other Basidiomycetes. Numbers at the base of branches are the percent bootstrap support ≥50%, which is a measure of support for the branch. The scale bar represents the distance of one amino acid substitution per site; 186 aligned amino acid sites were analyzed using the LG+25γ substitution model implemented in RAxML version 7.3.1 (Stamatakis 2006; Stamatakis 2008), resulting in this tree with an optimized LnL of −20412.06. The parameter describing the γ-distributed amino acid substitution frequencies is α = 1.42. Sequence accession numbers and the position of *C. cinereus* Nbs1 relative to all other eukaryotic homologs are given in Figure S4.

To identify *C. cinereus nbs1*, we searched by tBLASTn with several fungal orthologs and then aligned 25 fungal orthologs of Nbs1 and used this alignment to further search the *C. cinereus* genome by PSI-BLAST. We identified 300 amino acids of a *C. cinereus* protein that aligned with the N-terminus of Nbs1 orthologs, and our phylogenetic analyses showed that the putative *C. cinereus* Nbs1 grouped with other fungal Nbs1 orthologs (Figure 1 and Figure S4).

**Figure 2:**
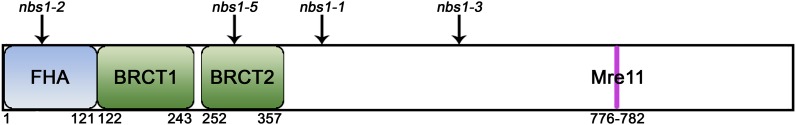
The template-based homology modeling program PHYRE was used to predict the location of functional domains within *C. cinereus* Nbs1. Numbers represent the amino acid boundaries of the functional domains. Locations of the predicted protein changes caused by *nbs1* mutations are represented by ↓. The *nbs1-2* mutation leads to an amino acid substitution, and *nbs1-1*, *nbs1-3*, and *nbs1-5* cause truncations. For *nbs1-1*, only the truncation at amino acid 406 is shown.

Using the predicted gene sequence to design primers, we amplified and sequenced *nbs1* from DNA and cDNA of a wild-type strain. *C. cinereus nbs1* is 3528 bp long and has 10 inferred introns, and the coding region is 3000 bp. The predicted protein has 999 amino acids and a predicted molecular weight of 110 kD (Figure 2). Using the template-based homology-modeling program PHYRE (Kelley and Sternberg 2009), we found that *C. cinereus* Nbs1 has the three conserved protein domains found in all other Nbs1 orthologs: an FHA domain and two tandem BRCT domains (Figure 2, Figure S1, and Figure S2). Most of the amino acids that form the binding pocket of the FHA domain, those that comprise the FHA/BRCT1 interface, and those that contact phosphorylated residues are precisely conserved or have conservative changes (Figure S1 and Figure S2). The C-terminus of the protein is predicted to be disordered, although a BLAST alignment between human Nbs1 and *C. cinereus* Nbs1 shows that the Mre11-binding peptide motif and the 100 amino acids surrounding it are highly conserved in *C. cinereus* (Figure S3). This region has 25% sequence identity and 40% similarity with human Nbs1. The exact Mre11 binding motif (NFKKFKK) is almost 100% identical to that of the human protein, except the last lysine is substituted with arginine. We were unable to identify an ATM-binding motif.

### Identification of *C. cinereus rad3* as Nbs1

Because two members of a previously characterized epistasis group required for DNA repair and meiosis (Valentine *et al.* 1995) were identified as *mre11* and *rad50*, respectively (Gerecke and Zolan 2000; Acharya *et al.* 2008), we tested the hypothesis that another member of that epistasis group, *rad3*, encodes the third member of the MRN complex, Nbs1. We sequenced *nbs1* in the *rad3* alleles and found mutations in each of the *rad3* alleles. The *rad3-1* has a mutation at bp 1235 (Table 1). It is an A→G in the 3′ splice site of the fifth intron. To determine how this mutation affects mRNA splicing, we used RT-PCR to amplify RNA from *rad3-1* mushroom caps and then cloned the PCR products. This showed that in 2 out of 23 clones that were sequenced, the fifth intron is not spliced out at all, leading to an immediate stop codon at the beginning of the intron and causing the predicted protein to truncate after amino acid 328. In the remaining clones, the intron was spliced at the next CAG, which is 40 bp downstream of the wild-type splice site. This leads to a stop codon that replaces the amino acid at 400, causing the predicted protein to truncate 55 amino acids after the second BRCT domain (Figure 2).

**Figure 3:**
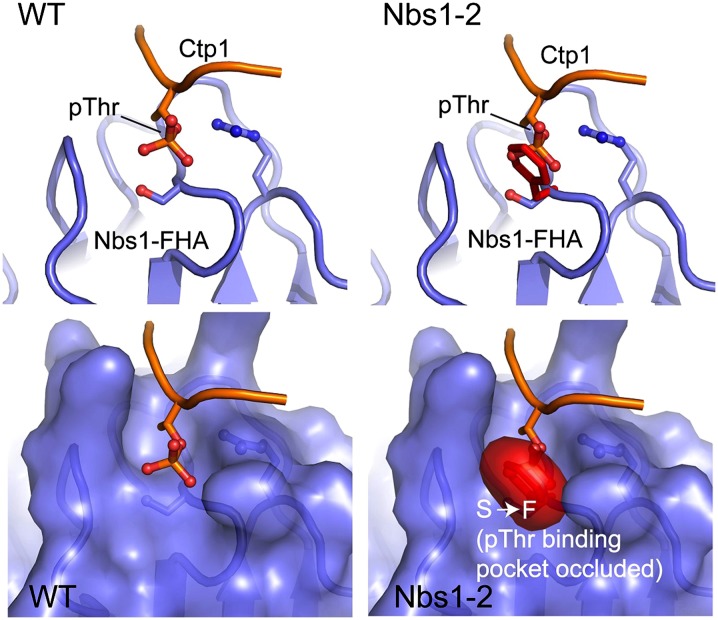
Predicted interaction between *C. cinereus* wild-type Nbs1 and Nbs1-2 protein with the phosphorylated threonine of Ctp1. *S. pombe* Nbs1 and *C. cinereus* Nbs1 were aligned using ClustalW and the *nbs1-2* mutation was mapped onto the *S. pombe* Nbs1 crystal structure. The phenylalanine likely occludes the binding pocked for Ctp1.

*rad3-2* has a CC→TT mutation at bp 188 and bp 189 that changes a serine to phenylalanine at amino acid 43 (Table 1 and Figure 2). Based on the crystal structure of Nbs1 in *S. pombe*, this serine is a critical residue for interaction with the phospho-threonine sites on Ctp1 (Williams *et al.* 2009). We mapped the *nbs1-2* mutation onto the crystal structure of *S. pombe* (Figure 3). It is predicted that the phenylalanine occludes the phospho-threonine binding pocket and disrupts interaction with Ctp1 at that site. Although no mutant data are available for *C. cinereus ctp1*, BLAST searches identify a putative ortholog containing a CtIP C-terminus domain (data not shown) (Marchler-Bauer *et al.* 2013).

*rad3-3* and *rad3-4* share the same mutation, a C→T at bp 2126 (Table 1). This mutation creates a stop codon at amino acid 570 (Figure 2). The predicted protein truncates 225 amino acids after the second BRCT repeat. *rad3-3* and *rad3-4* were isolated from the same screen; therefore, it is likely that they are the same mutant.

*rad3-5* is a complex mutation resulting from a 1-bp substitution at bp 1072 and a 1-bp deletion at 1074 (Table 1). This changes an alanine to valine at amino acid position 288 with a stop codon occurring immediately after. Therefore, the predicted protein is truncated in the middle of alpha helix 8 of the second BRCT domain (Figure 2).

To further test the hypothesis that *rad3* is *nbs1*, we transformed a phenotypically severe allele, *rad3-1*, with a wild-type copy of *nbs1*. Transformants were no longer radiation-sensitive and made viable spores. Because *nbs1* rescued *rad3-1* for both radiation sensitivity and meiotic defects, and because there are mutations in *nbs1* in all of the *rad3* alleles, we concluded that *rad3* is *nbs1*, and renamed the *C*. *cinereus rad3* gene *nbs1*.

### Meiotic nondisjunction and crossover control in *nbs1-2*

The *nbs1-2* mutation causes an amino acid change in the protein’s FHA domain of a residue known to be critical for phosphorylation-dependent protein–protein interactions (Figure 3). In *S. pombe*, the mutated residue interacts with Ctp1, a protein critical for DSB end resection, and in humans it interacts with Mdc1, which is a scaffolding protein required to localize the MRN complex to a break (Lloyd *et al.* 2009; Williams *et al.* 2009). The initial characterization of *nbs1-2* showed that it had a spore viability of 58% (*vs.* 83% in wild-type) and a hyper-recombination phenotype in one genetic interval (Zolan *et al.* 1988). Given that *nbs1-2* has a mutation in a domain critical for regulating the interaction of Nbs1 with other break repair proteins, and that there is both a recombination and spore viability defect, we tested the hypothesis that the observed *nbs1-2* phenotypes are caused by a defect in crossover control by determining whether the spore viability defect is attributable to meiotic nondisjunction and by measuring the rate and distribution of crossovers across an entire chromosome.

If crossover control is defective in *nbs1-2*, then there will likely be a higher incidence of meiosis I nondisjunction in this strain because achiasmate homologs will sometimes fail to segregate properly. To test for meiotic nondisjunction in *nbs1-2*, we isolated tetrads and asked how many spores of each tetrad were viable. We isolated 100 tetrads each from wild-type and *nbs1-2* crosses. The overall viabilities were 92.2% and 72.9% in wild-type and *nbs1-2*, respectively. The spore viability for *nbs1-2* was higher than that previously observed, likely because of differences in the methods used (Zolan *et al.* 1988). The distribution of tetrads containing 4, 3, 2, 1, or 0 viable spores was significantly different between wild-type and *nbs1-2* (*P* < 0.01, Fisher exact test) (Table 2). The proportion of tetrads with zero viable spores in wild-type was 1% and the proportion in *nbs1-2* was 16%, which is consistent with a higher frequency of meiosis I nondisjunction in *nbs1-2*. Tetrads with zero viable spores can occur if meiosis II nondisjunction occurs in both daughter cells, but this requires two independent rare events. It is statistically more likely that there was a single meiosis I nondisjunction event in the zero-viable spores class of *nbs1-2* tetrads.

**Table 2:** Number of viable scores per tetrad

Viability	Wild-Type (%)	*nbs1-2* (%)
4	78	62
3	13	11
2	7	12
1	0	4
0	1	17
Total viability	92.2	72.9

One hundred tetrads were counted for each strain. Distributions are significantly different (*P* < 0.01).

**Figure 4:**
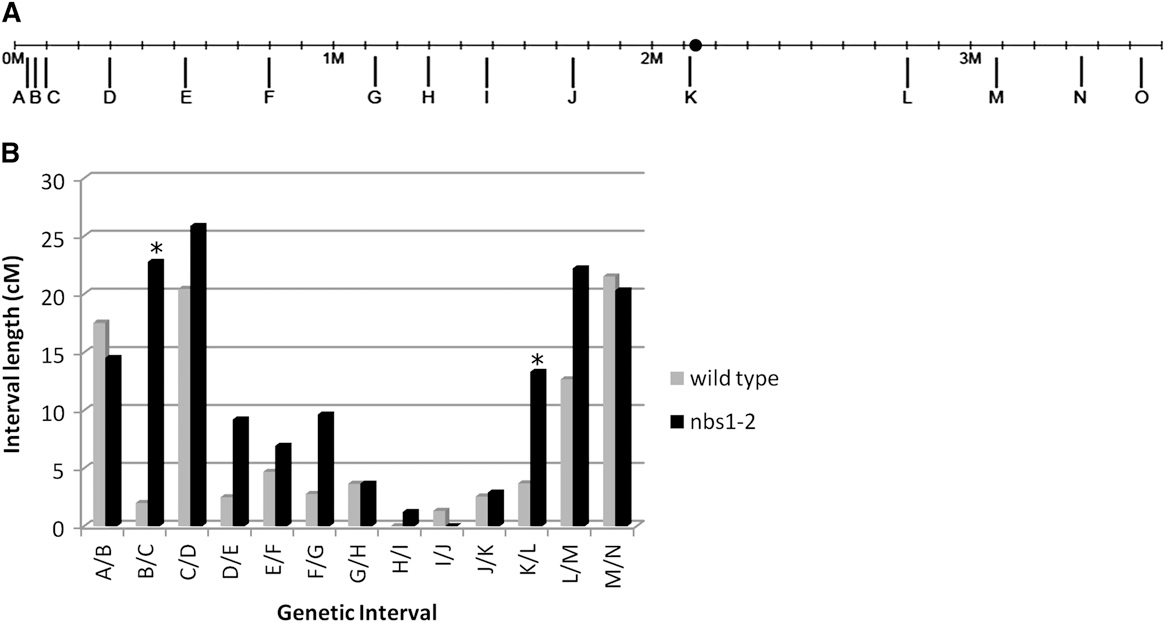
Genetic map of chromosome 3. (A) Location of SSRs, SNPs, and insertion-deletion polymorphisms used for genetic mapping of chromosome 3. Long tick marks below the line represent location of markers. The ● marks the centromere location (Stajich *et al.* 2010). (B) Map lengths of individual intervals along chromosome 3 of wild-type (gray) and *nbs1-2* (black). *Statistically significant intervals. Total map length is significantly different (*P* < 0.05).

**Figure 5:**
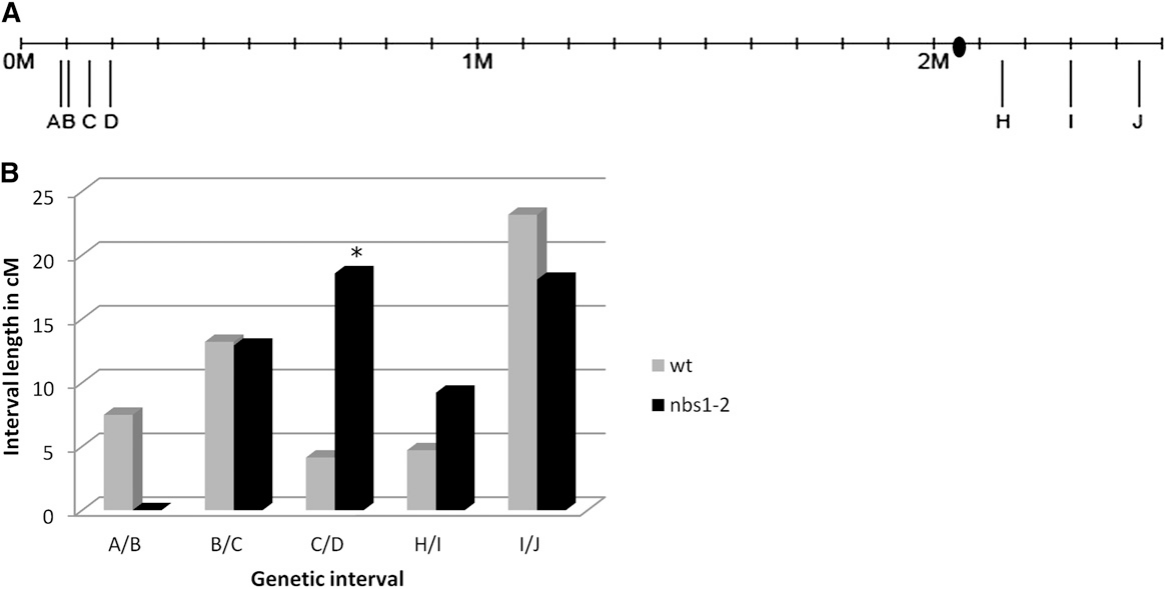
Genetic map of hotspots on chromosome 8. (A) Location of SSRs, SNPs, and insertion-deletion polymorphisms used for genetic mapping of chromosome 8. Long tick marks below the line represent location of markers. The ● marks the centromere location (Stajich *et al.* 2010). (B) Map lengths of hotspots on chromosome 8 of wild-type (gray) and *nbs1-2* (black). *Statistically significant intervals. Total map length is not significantly different.

Previous analysis of *nbs1-2* showed that there was an increase in the frequency of recombination in one genetic interval (Zolan *et al.* 1988). We further analyzed recombination in *nbs1-2* by using genetic mapping of simple sequence repeats, SNPs, and small insertion deletion polymorphisms to examine the frequency and distribution of crossovers across an entire chromosome (Figure 4 and Figure 5).

Genetic mapping of chromosome 3 showed an overall increase in crossover events from 95.4 cM in wild-type to 152.6 cM in *nbs1-2* (chi-square test, *P* < 0.01). The map lengths of intervals B/C and K/L are significantly different between wild-type and *nbs1-2* (B/C, *P* < 0.01; K/L, *P* < 0.05; Fisher exact test) (Figure 4). The distribution of crossovers across the chromosome was also significantly different between wild-type and *nbs1-2* (chi-square test with Monte Carlo simulation, 2000 samplings; *P* < 0.01). Previous cytological and genetic data showed that crossovers are largely subtelomeric in *C. cinereus* (Holm *et al.* 1981; Stajich *et al.* 2010). Our data confirm the previously noted chromosome 3 subtelomeric hotspots in intervals A/B, C/D, and M/N (Table 3). These hotspots in *nbs1-2* expand to include neighboring intervals. Additionally, some intervals that were cold for recombination in wild-type (>75 kb/cM) have genome average rates of recombination in *nbs1-2* (Table 3).

**Table 3:** Rates of recombination on chromosome 3

Interval	Wild-Type	*nbs1-2*
A/B	2.06	2.49
B/C	17.11	1.50
C/D	9.78	7.73
D/E	94.29	25.59
E/F	55.74	37.67
F/G	124.86	36.01
G/H	43.84	43.84
H/I	Linked	348.28
I/J	303.84	Linked
J/K	259.02	228.65
K/L	73.99	20.54
L/M	20.03	11.43
M/N	10.10	10.71

Data presented as kb/cM. Hotspots are <15 kb/cM, cold regions are >75 kb/cM, and average rates of recombination are between 15 and 75 kb/cM.

To test if the increase in recombination and spreading of hotspots occurs on other chromosomes in *nbs1-2*, we used genetic mapping of chromosome 8 hotspots (Figure 5). The total map length of chromosome 8 hotspots was not significantly different between wild-type and *nbs1-2* (52.7 cM in wild-type, 58.7 cM in *nbs1-2*; chi-square test, *P* = 0.16), even though there is one interval, C/D, that was significantly increased in *nbs1-2* (Figure 5). Despite no overall change in the number of crossovers in these hotspots, the distribution was significantly different between wild-type and *nbs1-2* (chi-square test with Monte Carlo simulation, 2000 samplings, *P* < 0.01) such that two hotspots were expanded to neighboring intervals and one was lost (Table 4).

**Table 4:** Rates of recombination on chromosome 8

Interval	Wild-Type	*nbs1-2*
A/B	4.10	Linked
B/C	3.38	3.46
C/D	12.24	2.72
H/I	27.41	14.05
I/J	7.26	9.31

Data presented as kb/cM. Hotspots are <15 kb/cM, cold regions are >75 kb/cM, and average rates of recombination are between 15 and 75 kb/cM.

To examine dominance of the crossover phenotype, we examined the crossover rate and distribution across chromosome 3 and the hotspots on chromosome 8 in a wild-type/*nbs1-2* heterokaryon. No intervals were significantly different when the heterokaryon was compared with the *nbs1-2* homokaryon, suggesting that the *nbs1-2* mutation is dominant for the crossover phenotype (Figure S5 and Figure S6).

Our sample size was not large enough to produce any statistically meaningful values of crossover interference (Table S6 and Table S7). However, there are significantly more double crossovers formed in *nbs1-2* than in wild-type (16 *vs.* 1, respectively; *P* < 0.01), suggesting some loss of crossover interference. The increase in double crossovers and increase in meiosis I nondisjunction (Table 2) suggest that crossover control is less effective in *nbs1-2*.

### DSB processing in *nbs1-2*

The *nbs1-2* mutation alters a site critical for interaction with Ctp1, and the bulky hydrophobic R group of phenylalanine, substituted for serine by mutation of the *nbs1* gene, is predicted to completely disrupt the Ctp1 interaction (Williams *et al.* 2009). Disruption of the binding ability of the FHA domain might affect DSB processing because the Ctp1–Nbs1 interaction is essential for initiating DSB end resection (Williams *et al.* 2009). We therefore decided to test the hypothesis that the formation and repair of meiotic DSBs are altered in *nbs1-2* by using immunolocalization to examine the appearance and disappearance of gamma-H2AX foci as a proxy for DSB formation and repair in wild-type and *nbs1-2*.

**Figure 6:**
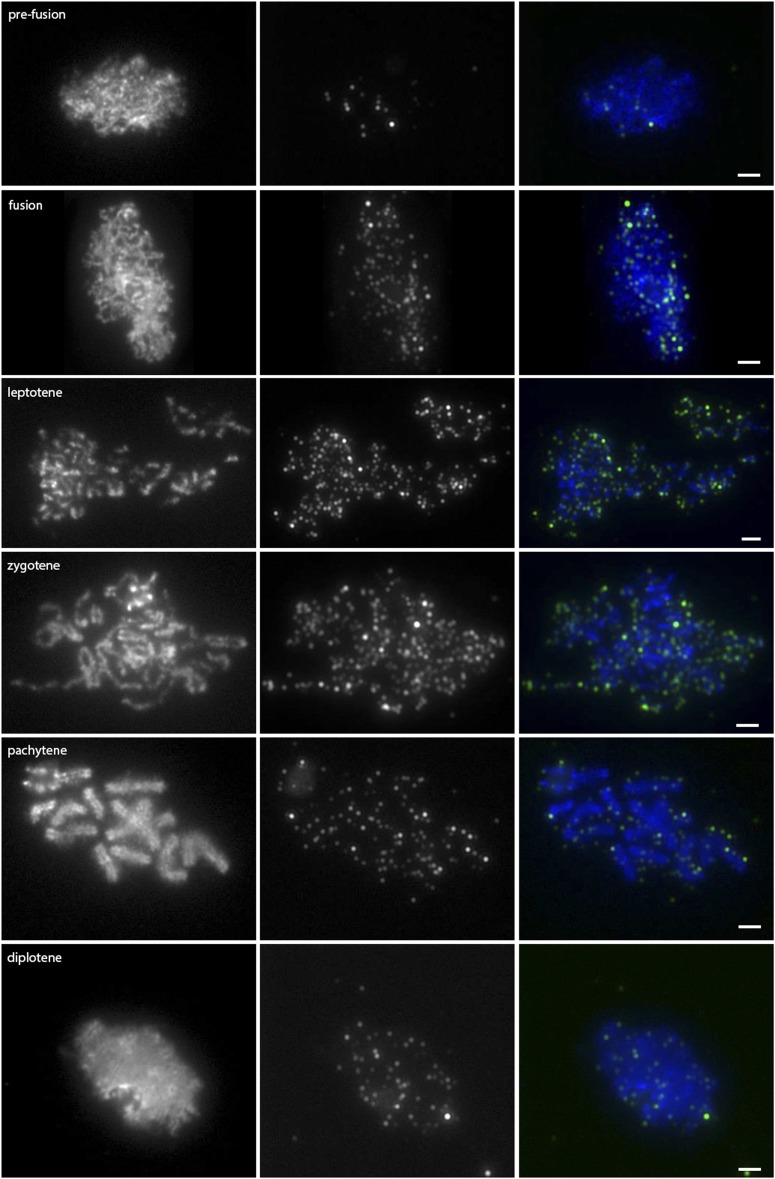
Anti-gamma-H2AX localization on wild-type meiotic chromosome spreads. Images in the left column are DAPI-stained chromosome spreads; the stage of prophase is indicated on the image. Images in the middle column are of anti-gamma-H2AX counterstained with a TRITC-conjugated secondary antibody, and images in the right column are the color-combined images. Prefusion, n = 42; leptotene, n = 47; zygotene, n = 50; pachytene, n = 48; diplotene, n = 60. Scale bars represent 2 µm.

A time course of wild-type meiotic chromosome spreads was stained with anti-gamma-H2AX to characterize wild-type DSB formation and repair during prophase I (Figure 6 and Figure 8). In prefusion (n = 42) and fusion nuclei (n = 13), there were low levels of gamma-H2AX staining; these foci likely represent a combination of background staining and DSBs formed during premeiotic replication. At leptotene (n = 47) and zygotene (n = 50), there was a dramatic induction of H2AX phosphorylation that remained through the end of pachytene (n = 48), disappearing during diffuse diplotene (n = 60) to levels lower than those in prefusion nuclei.

**Figure 7:**
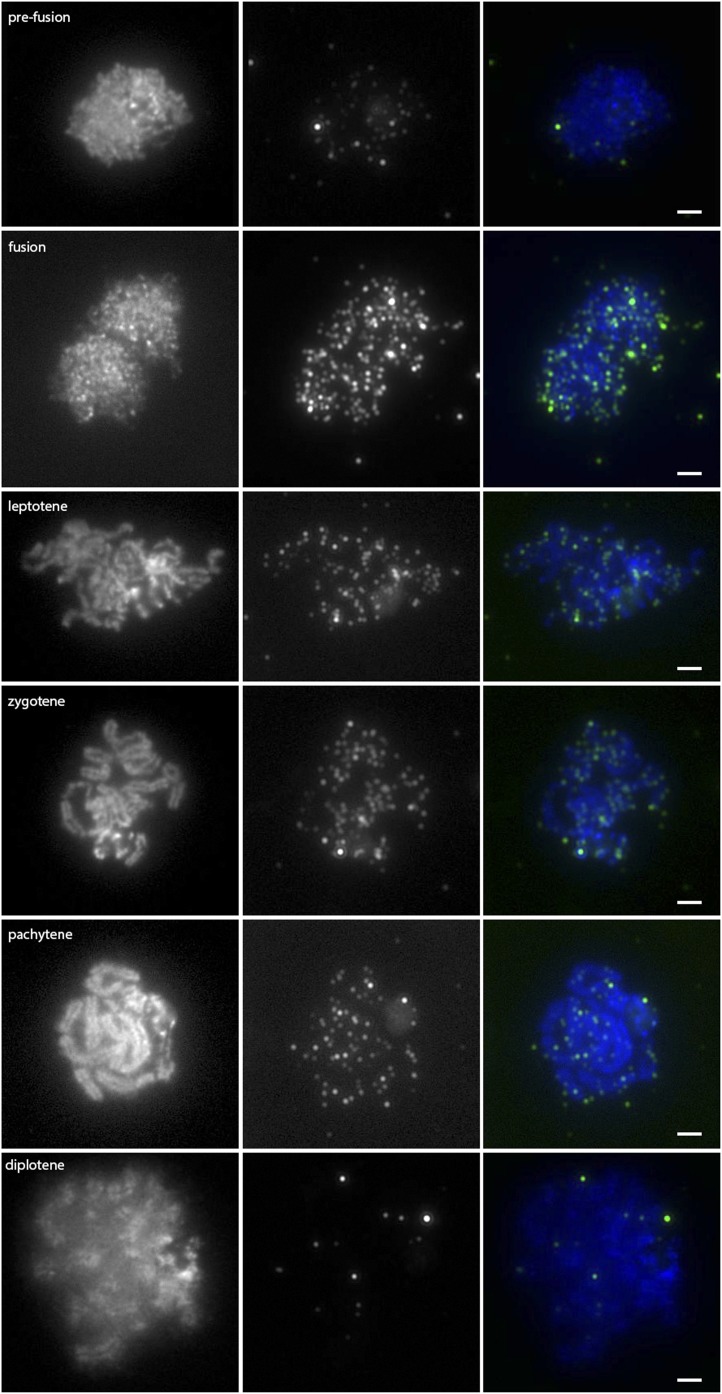
Anti-gamma-H2AX localization on *nbs1-2* meiotic chromosome spreads. Images in the left column are DAPI-stained chromosome spreads; the stage of prophase is indicated on the image. Images in the middle column are of anti-gamma-H2AX counterstained with a TRITC-conjugated secondary antibody, and images in the right column are the color-combined images. Prefusion, n = 38; leptotene, n = 46; zygotene, n = 34; pachytene, n = 49; diplotene, n = 39. Scale bars represent 2 µm.

**Figure 8:**
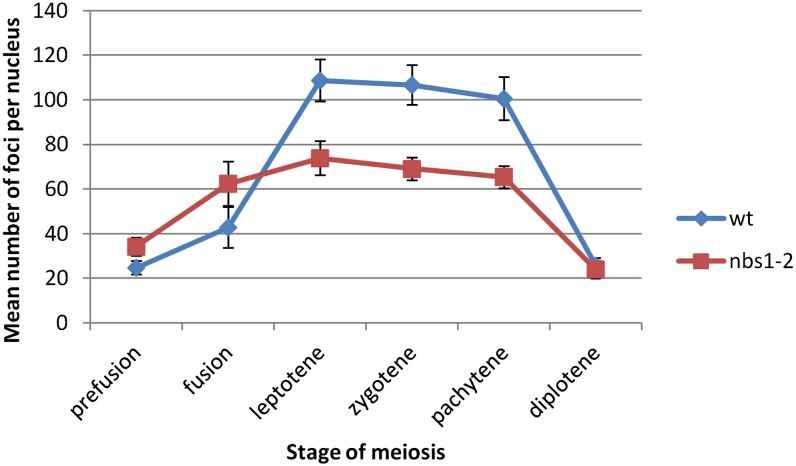
Time course of gamma-H2AX foci on meiotic chromatin spreads. Blue diamonds are wild-type and red squares are *nbs1-2*. Error bars represent 95% confidence intervals. Statistical analysis of both time courses was performed using a two-way ANOVA with Tukey multiple comparison test.

A time course of *nbs1-2* meiotic chromosome spreads was also stained with anti-gamma-H2AX (Figure 7 and Figure 8). There were low levels of foci in prefusion nuclei (n = 38). The number of foci significantly increased at fusion (n = 19; *P* < 0.01). However, at leptotene (n = 46), the number of foci was the same as at fusion (*P* > 0.05) and the number of foci stayed constant until diplotene (n = 39) when it declined to levels lower than those during prefusion.

At leptotene, zygotene, and pachytene, there were significantly more gamma-H2AX foci in wild-type than in *nbs1-2* (*P* < 0.01). However, levels of gamma-H2AX staining were the same in prefusion, fusion, and diplotene nuclei from wild-type and *nbs1-2* (*P* > 0.05) (Figure 8).

### *nbs1-2* is competent to phosphorylate H2AX in response to DSBs

**Figure 9:**
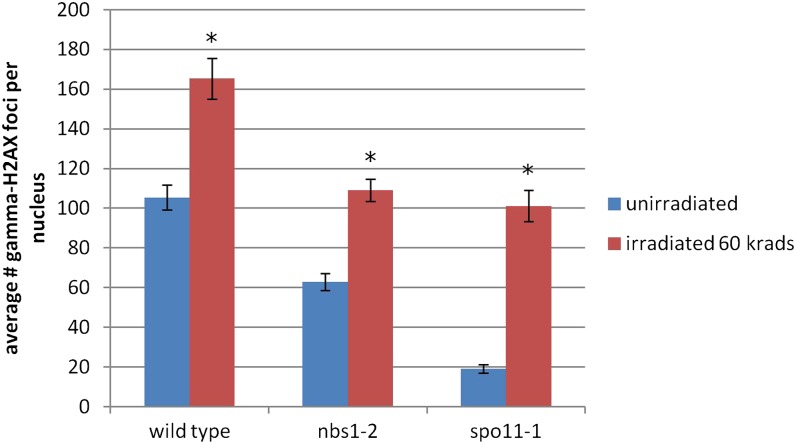
Average number of gamma-H2AX foci per nucleus from unirradiated mushrooms at 2 hr past karyogamy and 1 hr after irradiation with 60 krad. Error bars are 95% confidence intervals. There are significantly more gamma-H2AX foci in irradiated mushrooms for each genotype (Student *t* test, *P* < 0.01).

The lower number of gamma-H2AX foci in *nbs1-2* prophase nuclei suggested a defect in DSB formation, more rapid repair of DSBs, or a defect in H2AX phosphorylation. In the S-phase checkpoint response and in meiosis, Nbs1 activates ATM, and ATM then performs a wide variety of functions, including phosphorylating H2AX (Burma *et al.* 2001). We do not predict that the *nbs1-2* mutation affects binding to ATM; however, it is possible that there is some effect on ATM activation if Nbs1 cannot localize efficiently to the DSB. To ask if H2AX can be phosphorylated with wild-type kinetics in *nbs1-2*, we irradiated wild-type and *nbs1-2* with ionizing radiation 2 hr after the beginning of meiosis and then stained nuclei with anti-gamma-H2AX. We found a significant increase in the number of gamma-H2AX foci 1 hr after irradiation in both wild-type and *nbs1-2* (*P* < 0.01) (Figure 9). There was a similar increase in the number of foci in wild-type and *nbs1-2* (62% and 73% increases relative to unirradiated, respectively), indicating that H2AX is phosphorylated in response to DSBs in *nbs1-2* at a level similar to that in wild-type.

### H2AX phosphorylation is not dependent on Spo11 in *nbs1-2*

In wild-type, there is a significant increase in the number of gamma-H2AX foci from fusion to leptotene, implying that Spo11 has made DSBs (Figure 8). In *nbs1-2*, however, there was no statistical increase in gamma-H2AX foci between fusion and leptotene (Figure 8). To test if gamma-H2AX foci in *nbs1-2* are Spo11-dependent, we examined gamma-H2AX foci in a *spo11-1;nbs1-2* double mutant.

In mouse, H2AX can be phosphorylated independently of Spo11 activity (Chicheportiche *et al.* 2007). To test if this occurs in *C. cinereus*, we stained *spo11-1* meiotic nuclei at 2 hr past the beginning of meiosis (n = 31) (Figure S7). *spo11-1* is likely a null allele and nuclei do not progress past leptotene before undergoing programmed cell death; we chose to analyze nuclei 2 hr after the beginning of meiosis because this time point corresponds to leptotene in a wild-type nucleus (Celerin *et al.* 2000). There was an average of 19 gamma-H2AX foci per nucleus, a number that is similar to that of wild-type prefusion nuclei (Figure 6 and Figure 8). The low number of gamma-H2AX foci in *spo11-1* could represent Spo11-independent phosphorylation of H2AX, but we do not believe this affects our final analysis because these foci represent a small portion of the total number of foci. The *spo11-1* strain is capable of forming gamma-H2AX foci; after irradiation, there was a significant increase in the number of foci in the *spo11-1* strain examined (Figure 9).

**Figure 10:**
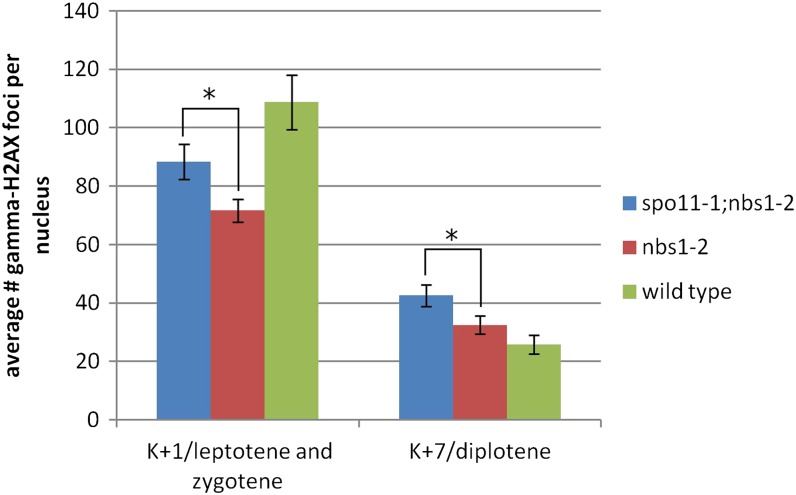
Average number of gamma-H2AX foci per nucleus from wild-type, *nbs1-2*, and *spo11-1;nbs1-2*. Because *spo11-1;nbs1-2* does not proceed through the typical meiotic prophase stages, nuclei from *spo11-1;nbs1-2* at 1 hr past karyogamy were compared to leptotene and zygotene nuclei of wild-type and *nbs1-2*. Nuclei from *spo11-1;nbs1-2* at 7 hr past karyogamy were compared to diplotene nuclei from wild-type and *nbs1-2*. Error bars are 95% confidence intervals.

To test whether H2AX phosphorylation is dependent on Spo11 in *nbs1-2*, we generated a *spo11-1;nbs1-2* double mutant and stained early and late meiotic nuclei (1 hr and 7 hr after the beginning of meiosis). The number of gamma-H2AX foci in *nbs1-2* is not greater than that in *spo11-1;nbs1-2* (Figure 10), as would be the case if gamma-H2AX foci were dependent on Spo11 in *nbs1-2*.

### *nbs1-2* has a replication defect

**Figure 11:**
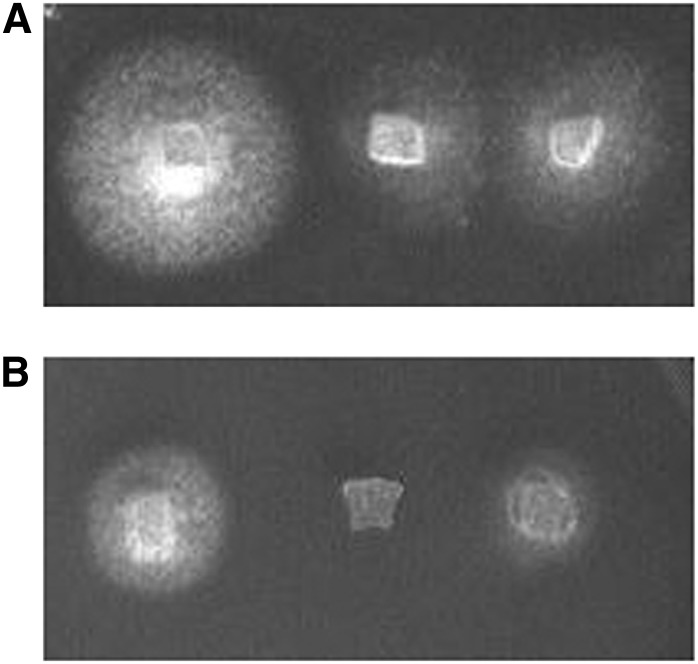
*nbs1-2* is sensitive to camptothecin. (A) Shown from left to right are wild-type, *nbs1-1*, and *nbs1-2* grown on mock-treated minimal medium after 2 d. (B) Shown from left to right are wild-type, *nbs1-1*, and *nbs1-2* grown on minimal medium containing 1 μM camptothecin after 2 d. Strains were scored for growth or no growth after 2 d.

Because the entire MRN complex is required to repair stalled replication forks and for fork protection, the gamma-H2AX foci in *nbs1-2* likely represent unrepaired breaks formed during premeiotic replication. To determine if *nbs1-2* has a replication defect, we tested whether it is sensitive to camptothecin, a DNA-damaging agent that causes DSBs at replication forks by preventing removal of topoisomerase I (Figure 11). Wild-type shows slower growth on medium containing 1 µM camptothecin than on medium that was mock-treated. *nbs1-1*, which is a more severe allele, shows no growth in the presence of camptothecin. However, *nbs1-2* shows an intermediate phenotype in that it is able to grow in the presence of camptothecin, but at a rate slower than that of wild-type (Figure 11). Further evidence of a mitotic replication defect in *nbs1-2* is our finding that only 37.8% of *nbs1-2* oidia, haploid spores that are the products of mitotic division during vegetative growth, contained nuclear DNA, whereas 89.2% of wild-type oidia contained nuclear DNA (*P* < 0.01).

## Discussion

### Conservation of *C. cinereus* Nbs1

Nbs1 is a highly diverged protein. In fact, when Nbs1 was initially identified as part of the human MRN complex, there was only weak evidence for homology to Xrs2 in *S. cerevisiae* (Carney *et al.* 1998). Thus, it was difficult to immediately identify *C. cinereus* Nbs1, even with the complete genome sequence. It was necessary to align many previously known fungal orthologs of Nbs1 and then use this alignment to analyze the *C. cinereus* genome. Even then, only the most conserved portions of Nbs1, the N-terminal FHA and first BRCT domains, were identified.

The FHA domain, the two tandem BRCT domains, and the Mre11 binding motif are well-conserved in *C. cinereus* Nbs1. Interestingly, as in *S. pombe*, *C. cinereus* Nbs1 is missing the phosphoprotein interaction site located in the BRCT domains, suggesting that loss of this interaction site is common to fungi (data not shown) (Williams *et al.* 2009). The crystal structure of *S. pombe* Nbs1 and the PHYRE analysis of *C. cinereus* Nbs1 indicate that the sequence C-terminal to the FHA and BRCT domains is highly disordered. It has been proposed that this part of the protein serves as a flexible tether between the C-terminus and N-terminus (Williams *et al.* 2009). If the function of this region is to serve as a tether, then the sequence identity of those particular amino acids may be flexible, allowing for divergence of this portion of the protein.

### Predicted effects of *nbs1* mutations

Because the *nbs1-1*, *nbs1-3*, and *nbs1-5* mutations cause the encoded proteins to truncate upstream of the predicted Mre11 binding domain (Figure 2), it might be predicted that the phenotype of those three mutants would be similar to that of *mre11-1*, which is a null allele (Gerecke 2000). However, fluorescence microscopy of gill segments on *nbs1-1* and *nbs1-3* showed very different phenotypes of chromatin condensation from each other and from *C. cinereus mre11-1* (Valentine *et al.* 1995; Zolan *et al.* 1995; Gerecke and Zolan 2000). Nbs1 might function differently in these two strains and their phenotypes might result from defects other than, or in addition to, a defect in bringing Mre11 into the nucleus. In fact, *nbs1-3* truncates 221 amino acids after the second BRCT domain, presumably leaving the N-terminal functional domains intact. This portion of the protein can be stably expressed in *E. coli* (Williams *et al.* 2009); in mammalian cells, irradiation-induced foci still form when the FHA and first BRCT domain are expressed alone, indicating that this part of the protein can still function in the absence of the C-terminus (Desai-Mehta *et al.* 2001). Together, these data suggest that *C. cinereus nbs1-3* may stably express the N-terminal portion of Nbs1, and that it may have some function in the cell. Although Nbs1-1 protein is predicted to truncate after the FHA and BRCT domains as well, the 49 amino acids remaining after the domain may not be enough to keep the protein fragment stable. For Nbs1-5, the protein truncates in the middle of the second BRCT domain. There are hydrophobic patches on the interior of the FHA-BRCT1-BRCT2 fragment in *S. pombe*, and exposure of those patches leads to an unstable protein (Williams *et al.* 2009). Therefore, in the Nbs1-5 protein fragment, it is likely the hydrophobic patches are exposed, causing the protein to be unstable. The *nbs1-5* strain may thus represent an *nbs1*-null phenotype.

The *nbs1-2* mutation causes an amino acid substitution in the FHA domain, changing a serine to a phenylalanine at amino acid 43. This serine comprises part of the binding pocket and directly contacts the phospho-threonine on Ctp1 (Williams *et al.* 2009). Modeling the mutation onto the Nbs1 crystal structure predicts that the bulky phenylalanine will occlude part of the phospho-threonine binding pocket and should alter Ctp1–Nbs1 interactions (Figure 3). Although the FHA and BRCT domains of Nbs1 provide architectural scaffolding to recruit proteins to a DSB, there is also remarkable structural flexibility within the protein. In *S. pombe*, Ctp1 binding to the FHA domain flips an arginine switch, which causes conformational changes in the tandem BRCT domains (Williams *et al.* 2009). It has been suggested that Nbs1 may exist in multiple conformational states depending on the microenvironment of a DSB (*e.g.*, what kind of DNA end Mre11 is bound to, what proteins are available for Nbs1 binding, cell-cycle stage), and that the conformational state of Nbs1, in conjunction with the conformational states of Mre11 and Rad50, may dictate the repair fate of a DSB (Williams *et al.* 2010). Thus, the *nbs1-2* mutation may alter the way in which proteins bind to the phosphoprotein binding cleft of the FHA domain, which in turn may either prevent the conformational changes associated with Ctp1 binding or inappropriately transmit information regarding the binding state of the protein to the BRCT domains. Either of these possibilities could cause severe downstream effects on DSB repair.

If the *nbs1-2* mutation does alter Ctp1 interaction with the binding pocket of the FHA domain, then the *nbs1-2* phenotype should mimic the phenotype of *S. pombe nbs1-R27A* or *K45A* mutants, both of which mutate one of the two sites of interaction with Ctp1 (Williams *et al.* 2009). In both *nbs1-R27A* and *K45A*, the strength of the interaction with Ctp1 is weakened eight-fold to 10-fold, but the DNA repair defect is apparent only at high concentrations of DNA-damaging agents (Lloyd *et al.* 2009; Williams *et al.* 2009). As predicted, *C. cinereus nbs1-2* is only mildly sensitive to high doses of ionizing radiation and to camptothecin (Zolan *et al.* 1988) (Figure 11).

### Crossovers in *nbs1-2* likely initiate from unrepaired DSBs formed during premeiotic replication

The pattern of DSB formation and repair in *nbs1-2* is strikingly different from that in wild-type (Figure 8); the number of DSBs in *nbs1-2* increases at nuclear fusion but does not further increase at leptotene, whereas wild-type nuclei show no increase in foci at nuclear fusion and undergo a significant increase in foci at leptotene. In addition, *nbs1-2* has enhanced sensitivity to camptothecin (Figure 11) and a defect in the production of oidia. These observations suggest that *nbs1-2* is unable to repair DSBs formed during replication and that these DSBs are the only, or the principal, source of DSBs used for meiotic crossovers. We have previously shown that in *C. cinereus*, irradiation partially rescues a *spo11-1* mutant for progression through meiosis and increases the spore viability (Celerin *et al.* 2000), showing that DSBs arising from DNA damage, rather than Spo11, can serve as substrates for meiotic crossovers in *C. cinereus*, as had been observed previously for other organisms (Sun *et al.* 1989; Dernburg *et al.* 1998; McKim *et al.* 2002). Interestingly, in *S. pombe* and *Caenorhabditis elegans*, dU:dG mismatches, which are a result of cytosine deamination, can partially rescue a *spo11* mutant (Pauklin *et al.* 2009). Cytosine deamination to uracil results in base excision repair-mediated breaks initiated by uracil DNA glycosylase (Hitomi *et al.* 2007), suggesting that DNA damage other than directly formed DSBs can serve as recombination substrates potentially because of breaks made during other repair processes.

The DSBs used for crossover formation in *nbs1-2* may originate from altered MRN functions at replication forks. Mre11 can bind and protect or process both replication forks and DSBs (Williams *et al.* 2008). At stalled replication forks in human cells, DNA-end processing or end protection by Mre11 depends on protein partners, including breast cancer type 2 susceptibility protein (BRCA2) and Fanconi anemia (FA) proteins BRCA1 and FANCD1 (Schlacher *et al.* 2011; Schlacher *et al.* 2012). These observations suggest that protection and/or processing of forks is a critical aspect of Mre11-Rad50-Nbs1 activities in avoiding DSBs at stalled replication forks. Given the impact of the Nbs1 FHA domain mutation shown here in increasing the number of DSBs present after meiotic DNA replication, it may be useful to evaluate the roles of Nbs1 in modulating Mre11 activity in replication fork protection and processing pathways.

The observation that damage caused during replication can rescue defects in meiotic recombination is not unprecedented. In *S. pombe*, a mutation in the flap nuclease FEN-1 can rescue the meiotic phenotype of a *rec12* mutant (the *S. pombe* ortholog of *spo11*) (Farah *et al.* 2005a). FEN-1 is thought to help process Okazaki fragments during DNA replication by precisely removing flaps to leave a product that can be directly ligated (Tsutakawa *et al.* 2011), therefore any lesions left unrepaired in a FEN-1 mutant may be the source of recombinogenic damage in a *fen-1 rec12* mutant. Also, in *S. pombe*, DSBs generated at a palindrome during premeiotic replication are partially dependent on the MRN complex and are repaired during meiotic recombination (Farah *et al.* 2005b).

*nbs1-2* did not show an increase in gamma-H2AX foci at leptotene, suggesting that Spo11 does not induce DSBs in this strain (Figure 8). Although replication itself is not required for Spo11-induced DSBs (Murakami and Nurse 2001; Hochwagen *et al.* 2005), signaling activities important for DSB formation, such as Mer2 phosphorylation by Cdc7 and CDK-S, do occur during the time of replication (Murakami and Keeney 2008). Given the connection between events that occur during replication and Spo11-induced DSB formation, it might be that the potential replication fork problems in *nbs1-2* disturb replication such that Spo11 activity is inhibited through a regulatory process. Alternatively, or in addition, the DSBs formed in *nbs1-2* during the DNA replication preceding meiosis could affect the state of *nbs1-2* chromatin in early meiosis such that it is refractive to Spo11 activity.

It might be expected that unrepaired DSBs formed during replication would activate checkpoints and prevent entry into meiosis. However, if the conformational state of the MRN complex can dictate its activity (Williams *et al.* 2010), and given that the complex is required for DNA damage checkpoint activation (Usui *et al.* 2001; Lee and Paull 2005), it is possible that *nbs1-2* escapes checkpoint activation because the mutant MRN complex is not in the right conformational state to turn on the checkpoint. Alternatively, there may be a threshold of damage required to prevent entry into meiosis and this threshold is not reached in *nbs1-2*.

A *spo11-1;nbs1-2* double mutant forms significantly more gamma-H2AX foci at leptotene/zygotene than an *nbs1-2* single mutant (Figure 10). Although we cannot rule out the possibility that the DSBs in the double mutant are of a different origin than those in the *nbs1-2* single mutant, *e.g.*, represent chromosome fragmentation in a dying cell, we have not observed early basidial cell death in other *spo11-1* double mutants (for example, *spo11-1;rad50* double mutants) (Acharya *et al.* 2008). Therefore, we think that the DSBs in the *spo11-1;nbs1-2* double mutant reflect the same premeiotic process that causes DSBs in the *nbs1-2* single mutant, *i.e.*, unrepaired DSBs formed during premeiotic replication. However, a *spo11-1;nbs1-2* double mutant does not make any viable spores (data not shown), whereas *nbs1-2* does. Therefore, Spo11 has at least one critical meiotic function in *nbs1-2*. Because there is no Spo11-dependent increase in gamma-H2AX foci in *nbs1-2* (Figure 10), this role is in something other than DSB formation. A role for Spo11 in the timing of premeiotic DNA replication and in DSB-independent homolog pairing has been reported for *S. cerevisiae* (Cha *et al.* 2000; Sharif *et al.* 2002; Boateng *et al.* 2013). In *C. cinereus*, *spo11* is maximally expressed during fusion, consistent with the idea that it may have a function during replication (Burns *et al.* 2010). In addition, *spo11* transcripts and protein are expressed during late prophase, indicating post-DSB functions as well (Bellani *et al.* 2010).

### Crossover control in *nbs1-2*

The crossover distribution in *nbs1-2* is altered relative to that in wild-type (Figure 4 and Figure 5). If we are correct that crossovers in *nbs1-2* originate from persistent premeiotic replication-dependent DSBs that are competent to form crossovers, then these crossovers are not subject to the crossover control mechanisms that bring about the normal distribution in *C. cinereus*, at least not to the same degree as the Spo11-induced breaks. It is possible that replication-dependent DSBs are repaired as noninterfering crossovers by a structure-specific nuclease such as Mus81, which has been shown to act at replication forks and to form noninterfering crossovers during meiosis (Chen *et al.* 2001; Kaliraman *et al.* 2001; Smith *et al.* 2003; Osman *et al.* 2003).

The lower numbers of DSBs in *nbs1-2* relative to wild-type at leptotene, zygotene, and pachytene could result from less DSB formation in *nbs1-2*; alternatively, *nbs1-2* could repair meiotic DSBs more quickly than wild-type. Our observation that late prophase cells in wild-type and *nbs1-2* have the same number of remaining gamma-H2AX foci supports the notion that the time course of meiotic DSB repair is similar in *nbs1-2* and wild-type. Therefore, it is likely that meiotic cells in *nbs1-2* convert a higher percentage of DSBs to crossovers than do wild-type cells.

Notably, although hotspots are larger in *nbs1-2* than in wild-type, the general subtelomeric location of hotspots is maintained (Figure 4 and Figure 5). Therefore, we infer that the repair pathway for a meiotic DSB in *C. cinereus* is determined, at least in part, by its location relative to the chromosome as a whole, *i.e.*, a subtelomeric DSB is likely to become a crossover, regardless of its origin. Alternatively, replication-induced DSBs could be formed or maintained preferentially in subtelomeric locations.

It is clear that there is both a spore viability defect and an increase in meiosis I nondisjunction in *nbs1-2*. The increased meiosis I nondisjunction could result from a proportion of cells in which smaller chromosomes lack DSBs and, hence, crossovers; it is likely that replication-induced breaks are not subject to crossover controls, and therefore their distribution among chromosomes is expected to be stochastic. Alternatively, or in addition, it is possible that the increase in meiosis I nondisjunction in *nbs1-2* is attributable to a higher incidence of double crossovers in subtelomeric regions. For a chiasma to be effective, the crossover must have a region distal to the exchange long enough to maintain the chiasma. It is possible that a double crossover shortens the length of the exchanged region, resulting in a weaker chiasma and also preventing the exchange region from continuing to the nuclear envelope. It has been suggested that interactions between the telomeres and the nuclear envelope help maintain chiasmata (Holm *et al.* 1981).

Our findings lead to the conclusion that Spo11-independent meiotic recombination, in the presence of wild-type Spo11 protein, can lead to viable spore production that is only slightly, albeit significantly, lower than that in wild-type. Crossover control, a signature and central part of meiosis, thus serves to increase the fidelity of chromosome segregation, but it is not required for basic execution of the meiotic process if DSB and crossover frequencies are high enough. Our study also suggests that premeiotic DSBs can be sufficient to support accurate meiotic chromosome segregation, and our work supports previous observations (Cha *et al.* 2000; Bellani *et al.* 2010) that Spo11 has at least one critical meiotic role independent of its function in creating meiotic DSBs.

## Supplementary Material

Supporting Information
