# Supplementary material for: A Mutation in the FHA Domain of Coprinus cinereus Nbs1 Leads to Spo11-Independent Meiotic Recombination and Chromosome Segregation
Source: G3 (Bethesda). 2013 Nov 1;3(11):1927–43. doi: 10.1534/g3.113.007906 (PMC3815056; doi:10.1534/g3.113.007906)
Supplement: Supporting Information [file supp_3_11_1927__index.html]

A Mutation in the FHA Domain of Coprinus cinereus Nbs1 Leads to Spo11-Independent Meiotic Recombination and Chromosome Segregation — Supporting Information 

# A Mutation in the FHA Domain of *Coprinus cinereus* Nbs1 Leads to Spo11-Independent Meiotic Recombination and Chromosome Segregation

## Supporting Information for Crown *et al.*, 2013

**Files in this Data Supplement:**

- Supporting Information - Figures S1-S7 and Tables S1-S7 (PDF, 1 MB)
- Figure S1 - PHYRE2 alignment of *C. cinereus* Nbs1 and *S. pombe* Nbs1 (Kelley and Sternberg 2009). (PDF, 439 KB)
- Figure S2 - PHYRE2 alignment of *C. cinereus* Nbs1 and human Bard1 BRCT tandem repeat (Kelley and Sternberg 2009). (PDF, 415 KB)
- Figure S3 - Alignment of the Mre11 binding motif (amino acids 770-776) and surrounding sequence from *C. cinereus* and human Nbs1. (PDF, 340 KB)
- Figure S4 - Phylogenetic tree showing the evolutionary relationships among eukaryotic orthologs of *nbs1* from animals, fungi, plants and protists. (PDF, 859 KB)
- Figure S5 - Map lengths of individual intervals along chromosome 3 from the homozygous and heterozygous nbs1-2 crosses. (PDF, 319 KB)
- Figure S6 - Map lengths of individual hotspot intervals on chromosome 8. (PDF, 317 KB)
- Figure S7 - anti-gamma-H2AX localization on *spo11-1* meiotic chromosome spreads from unirradiated mushrooms at 2 hours past karyogamy (n=31), and one hour (n=32) and three hours(n=32) after irradiation with 60 krads. (PDF, 450 KB)
- Table S1 - Primer sequences used to amplify *C. cinereus nbs1*. (PDF, 419 KB)
- Table S2 - Primers used to amplify simple sequence repeats on chromosomes 3 and 8. (PDF, 309 KB)
- Table S3 - Size of parental alleles used for genotyping. (PDF, 310 KB)
- Table S4 - Primers used to amplify single nucleotide polymorphisms on chromosomes 3 and 8. (PDF, 307 KB)
- Table S5 - Single nucleotide polymorphisms used for genetic mapping. (PDF, 307 KB)
- Table S6 - Interference on chromosome 3. (PDF, 418 KB)
- Table S7 - Interference among chromosome 8 hotspots. (PDF, 416 KB)
