# Supplementary material for: A Mutation in the FHA Domain of Coprinus cinereus Nbs1 Leads to Spo11-Independent Meiotic Recombination and Chromosome Segregation
Source: G3 (Bethesda). 2013 Nov 1;3(11):1927–43. doi: 10.1534/g3.113.007906 (PMC3815056; doi:10.1534/g3.113.007906)
Supplement: Supporting Information [file supp_g3.113.007906_007906SI.pdf]

G3 Genes | Genomes | Genetics

A mutation in the FHA domain of *Coprinus cinereus* Nbs1 leads to Spo11-independent meiotic recombination and chromosome segregation

K. Nicole Crown[*,1], Oleksandr P. Savytskyy[*], Shehre-Banoo Malik[**], John Logsdon[**], R. Scott Williams[§], John A. Tainer[§§], Miriam E. Zolan[*]

*Department of Biology, Indiana University, Bloomington, Indiana 47405
**Department of Biology, University of Iowa, Iowa City, Iowa 52242
§National Institute of Environmental Health Sciences, Research Triangle Park, North Carolina 27709
§§The Scripps Research Institute, La Jolla, California 92037
1Current address: Department of Biology, University of North Carolina at Chapel Hill, Chapel Hill, NC 27599

DOI: 10.1534/g3.113.007906

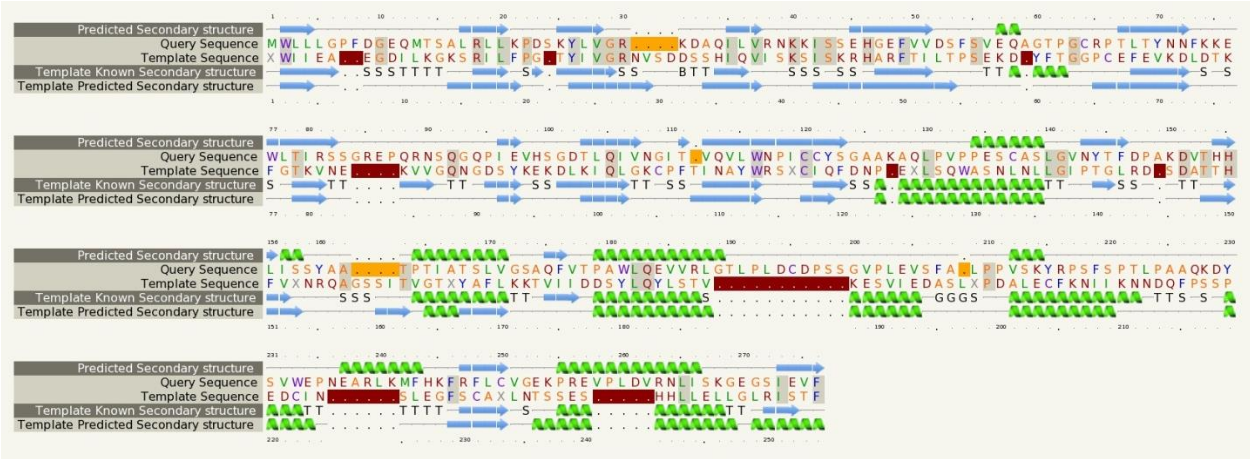

Figure S1 PHYRE2 alignment of *C. cinereus* Nbs1 and *S. pombe* Nbs1 (Kelley and Sternberg 2009). The query sequence is *C. cinereus* Nbs1 and the template sequence is *S. pombe* Nbs1. Predicted secondary structures are made by the PHYRE2 program, and the *S. pombe* known secondary structure is from the crystal structure (Williams et al. 2009). Blue arrows represent beta sheets and green spirals represent alpha helices. Amino acids highlighted in red represent insertions in the *C. cinereus* protein sequence relative to *S. pombe* and those highlighted in yellow represent deletions in the *C. cinereus* protein sequence relative to *S. pombe.* Amino acids 1-115 of *C. cinereus* Nbs1 correspond to the FHA domain of *S. pombe* Nbs1.

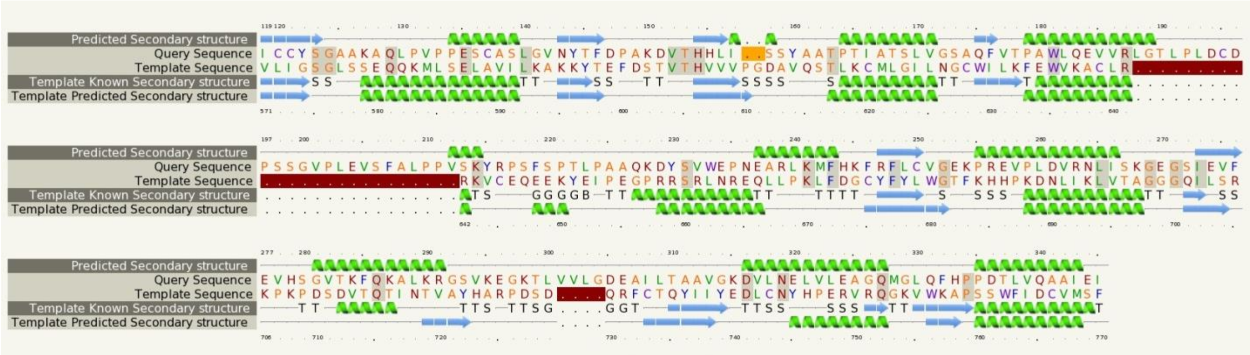

Figure S2 PHYRE2 alignment of *C. cinereus* Nbs1 and human Bard1 BRCT tandem repeat (Kelley and Sternberg 2009). The query sequence is *C. cinereue* Nbs1 and the template sequence is the human Bard1 BRCT tandem repeat . Predicted secondary structures are made by the PHYRE2 program, and human known secondary structure is from the crystal structure. Blue arrows represent beta sheets and green spirals represent alpha helices. Amino acids highlighted in red represent insertions in the *C. cinereus* protein sequence relative to human and those highlighted in yellow represent deletions in the *C. cinereus* protein sequence relative to human. The BRCT1 domain of *C. cinereus* Nbs1 is from amino acids 119-237 and the BRCT2 domain is from amino acids 246-345 of *C. cinereus*.

```
Coprinus    GKKREDEFDREFNRLKISKPDLDAQNGREPEEDWKVLADFGDDSGIRGNFMTICELEVFK  749
            GKKRE + D  ++  +IS  D                    DDS +    + + E
Human       GKKRELKEDSLWSAKEISNND-----------------KLQDDSEMLPKKLLLTEFRSLV  663

Coprinus    EKNGRNAKSAGEMRPEWEGKPNFKKFKRKNVPRSG  784
             KN  +   +G +  ++    NFKKFK+   P +G
Human       IKNSTSRNPSG-INDDYGQLKNFKKFKKVTYPGAG  697
```

Figure S3 Alignment of the Mre11 binding motif (amino acids 770-776) and surrounding sequence from *C. cinereus* and human Nbs1. The *C. cinereus* protein sequence was used as a query in a BLAST against the human protein sequence to produce an alignment.

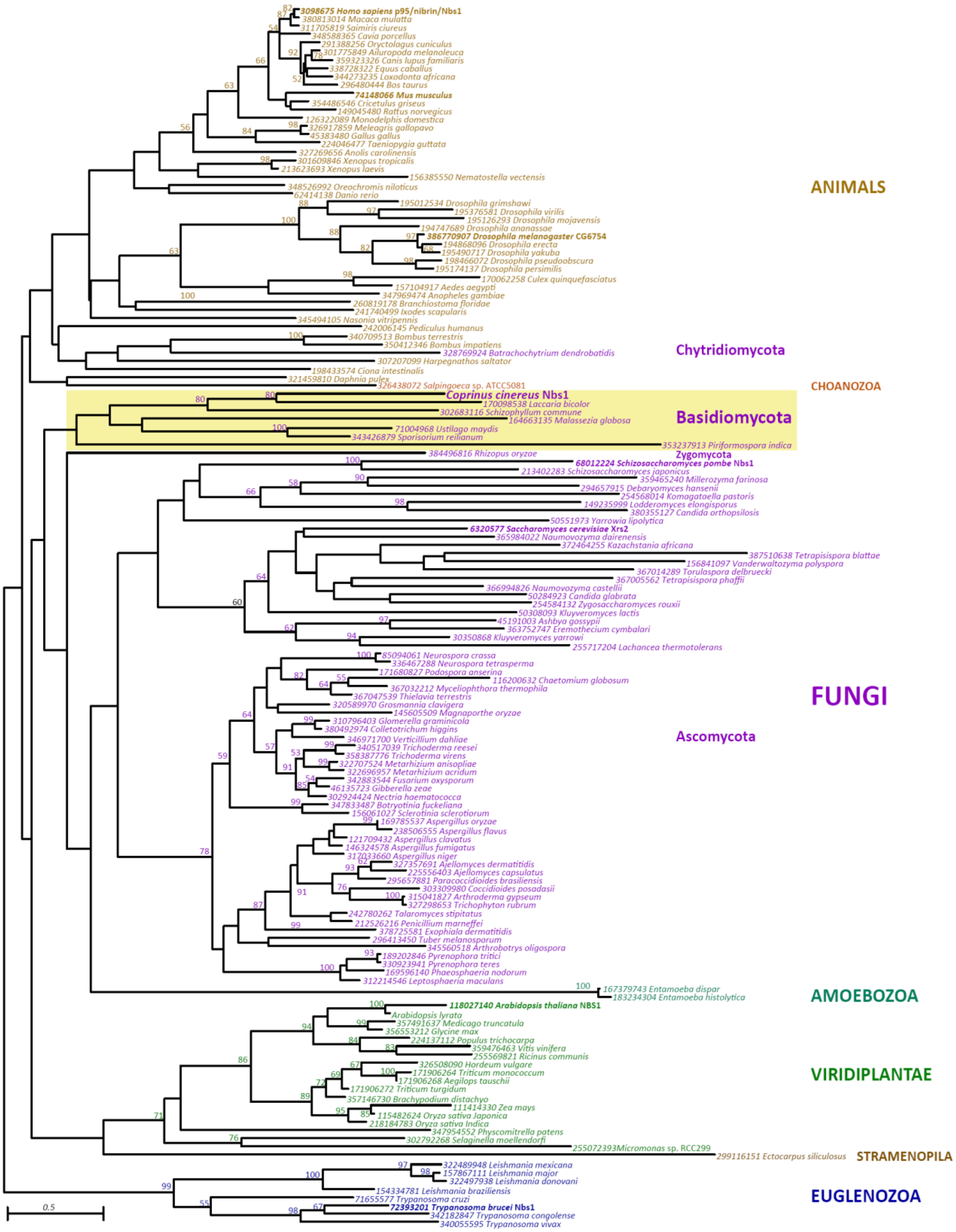
3098675 Homo sapiens p95/nibrin/Nbs1
380813014 Macaca mulatta
311705819 Saimiris ciureus
348588365 Cavia porcellus
291388256 Oryctolagus cuniculus
301775849 Ailuropoda melanoleuca
359323326 Canis lupus familiaris
338728322 Equus caballus
344273235 Loxodonta africana
296480444 Bos taurus
74148066 Mus musculus
354486546 Cricetulus griseus
149045480 Rattus norvegicus
126322089 Monodelphis domestica
326917859 Meleagris gallopavo
45383480 Gallus gallus
224046477 Taeniopygia guttata
327269656 Anolis carolinensis
301609846 Xenopus tropicalis
213623693 Xenopus laevis
156385550 Nematostella vectensis
348526992 Oreochromis niloticus
62414138 Danio rerio
ANIMALS
195012534 Drosophila grimshawi
195376581 Drosophila virilis
195126293 Drosophila mojavensis
194747689 Drosophila ananassae
386770907 Drosophila melanogaster CG6754
194868096 Drosophila erecta
195490717 Drosophila yakuba
198466072 Drosophila pseudoobscura
195174137 Drosophila persimilis
170062258 Culex quinquefasciatus
157104917 Aedes aegypti
347969474 Anopheles gambiae
260819178 Branchiostoma floridae
241740499 Ixodes scapularis
345494105 Nasonia vitripennis
242006145 Pediculus humanus
340709513 Bombus terrestris
350412346 Bombus impatiens
328769924 Batrachochytrium dendrobatidis
Chytridiomycota
307207099 Harpegnathos saltator
198433574 Ciona intestinalis
321459810 Daphnia pulex
326438072 Salpingoeca sp. ATCC5081
CHOANOZOA
Coprinus cinereus Nbs1
170098538 Laccaria bicolor
302683116 Schizophyllum commune
164663135 Malassezia globosa
Basidiomycota
71004968 Ustilago maydis
343426879 Sporisorium reilianum
353237913 Piriformospora indica
384496816 Rhizopus oryzae
Zygomycota
68012224 Schizosaccharomyces pombe Nbs1
213402283 Schizosaccharomyces japonicus
359465240 Millerozyma farinosa
294657915 Debaryomyces hansenii
254568014 Komagataella pastoris
149235999 Lodderomyces elongisporus
380355127 Candida orthopsilosis
50551973 Yarrowia lipolytica
6320577 Saccharomyces cerevisiae Xrs2
365984022 Naumovozyma dairenensis
372464255 Kazachstania africana
387510638 Tetrapisispora blattae
156841097 Vanderwaltozyma polyspora
367014289 Torulaspora delbrueckii
367005562 Tetrapisispora phaffii
366994826 Naumovozyma castellii
50284923 Candida glabrata
254584132 Zygosaccharomyces rouxii
50308093 Kluyveromyces lactis
45191003 Ashbya gossypii
363752747 Eremothecium cymbalari
30350868 Kluyveromyces yarrowi
255717204 Lachancea thermotolerans
85094061 Neurospora crassa
336467288 Neurospora tetrasperma
171680827 Podospora anserina
116200632 Chaetomium globosum
367032212 Myceliophthora thermophila
367047539 Thielavia terrestris
320589970 Grosmannia clavigera
145605509 Magnaporthe oryzae
FUNGI
310796403 Glomerella graminicola
380492974 Colletotrichum higgins
Ascomycota
346971700 Verticillium dahliae
340517039 Trichoderma reesei
358387776 Trichoderma virens
322707524 Metarhizium anisopliae
322696957 Metarhizium acridum
342883544 Fusarium oxysporum
46135723 Gibberella zeae
302924424 Nectria haematococca
347833487 Botryotinia fuckeliana
156061027 Sclerotinia sclerotiorum
169785537 Aspergillus oryzae
238506555 Aspergillus flavus
121709432 Aspergillus clavatus
146324578 Aspergillus fumigatus
317033660 Aspergillus niger
327357691 Ajellomyces dermatitidis
225556403 Ajellomyces capsulatus
295657881 Paracoccidioides brasiliensis
303309980 Coccidioides posadasii
315041827 Arthroderma gypseum
327298653 Trichophyton rubrum
242780262 Talaromyces stipitatus
212526216 Penicillium marneffei
378725581 Exophiala dermatitidis
296413450 Tuber melanosporum
345560518 Arthrobotrys oligospora
189202846 Pyrenophora tritici
330923941 Pyrenophora teres
169596140 Phaeosphaeria nodorum
312214546 Leptosphaeria maculans
167379743 Entamoeba dispar
183234304 Entamoeba histolytica
AMOEBOZOA
118027140 Arabidopsis thaliana NBS1
Arabidopsis lyrata
357491637 Medicago truncatula
356553212 Glycine max
224137112 Populus trichocarpa
359476463 Vitis vinifera
255569821 Ricinus communis
326508090 Hordeum vulgare
171906264 Triticum monococcum
171906268 Aegilops tauschii
171906272 Triticum turgidum
357146730 Brachypodium distachyo
111414330 Zea mays
115482624 Oryza sativa Japonica
218184783 Oryza sativa Indica
347954552 Physcomitrella patens
302792268 Selaginella moellendorfi
255072393Micromonas sp. RCC299
VIRIDIPLANTAE
299116151 Ectocarpus siliculosus
STRAMENOPILA
322489948 Leishmania mexicana
157867111 Leishmania major
322497938 Leishmania donovani
154334781 Leishmania braziliensis
71655577 Trypanosoma cruzi
72393201 Trypanosoma brucei Nbs1
342182847 Trypanosoma congolense
340055595 Trypanosoma vivax
EUGLENOZOA
0.5

Figure S4 Phylogenetic tree showing the evolutionary relationships among eukaryotic orthologs of *nbs1* from animals, fungi, plants and protists*. C. cinereus* Nbs1 groups with its closest relative *Laccaria* and other Basidiomycete fungi. Branch support is indicated by numbers at the base of branches, the percent boostrap support $\geq$ 50%. The scale bar represents the distance of 0.5 amino acid substitutions per site. 186 aligned amino acid sites were analyzed using the LG+25γ substitution model implemented in RAxML version 7.3.1*,* resulting in this tree with an optimized LnL=-41564.91. The parameter describing the γ-distributed amino acid substitution frequencies is α=1.58. The GenInfo Identifier numbers for each sequence from Genbank are indicated.

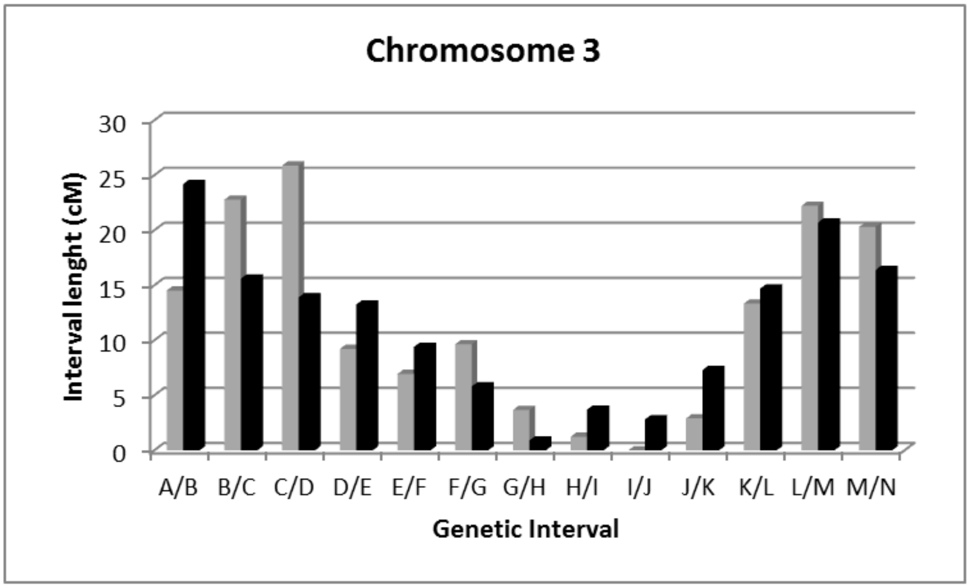

Figure S5 Map lengths of individual intervals along chromosome 3 from the homozygous and heterozygous nbs1-2 crosses. Grey bars are the homokaryon nbs1-2 cross and black bars are the heterokaryon wild type x nbs1-2 cross. No interval is statistically significantly different, nor is the total map lengths.

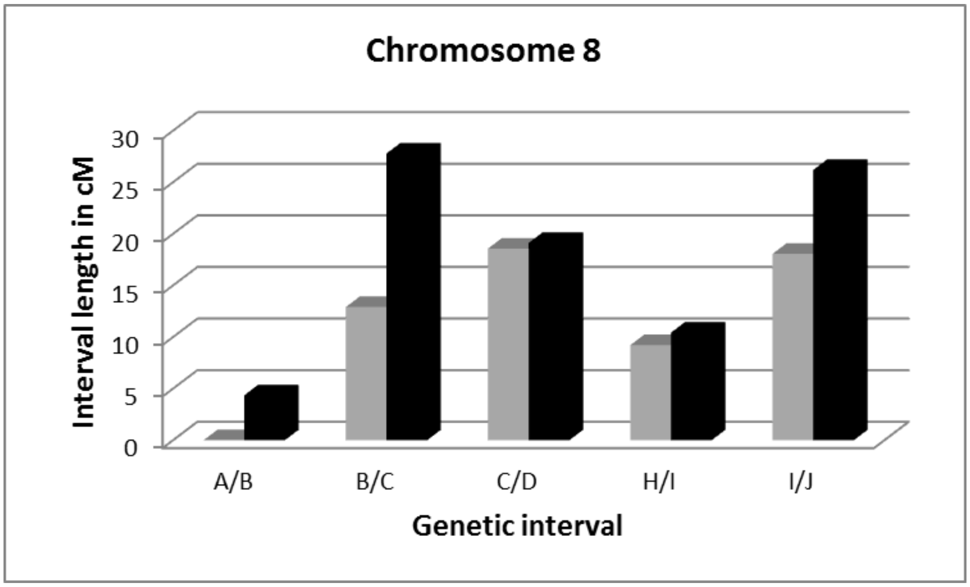

Figure S6 Map lengths of individual hotspot intervals on chromosome 8. Light grey bars are the homokaryon nbs1-2 cross and black bars are the heterokaryon wild type x nbs1-2 cross. Total map lengths are not statistically significantly different.

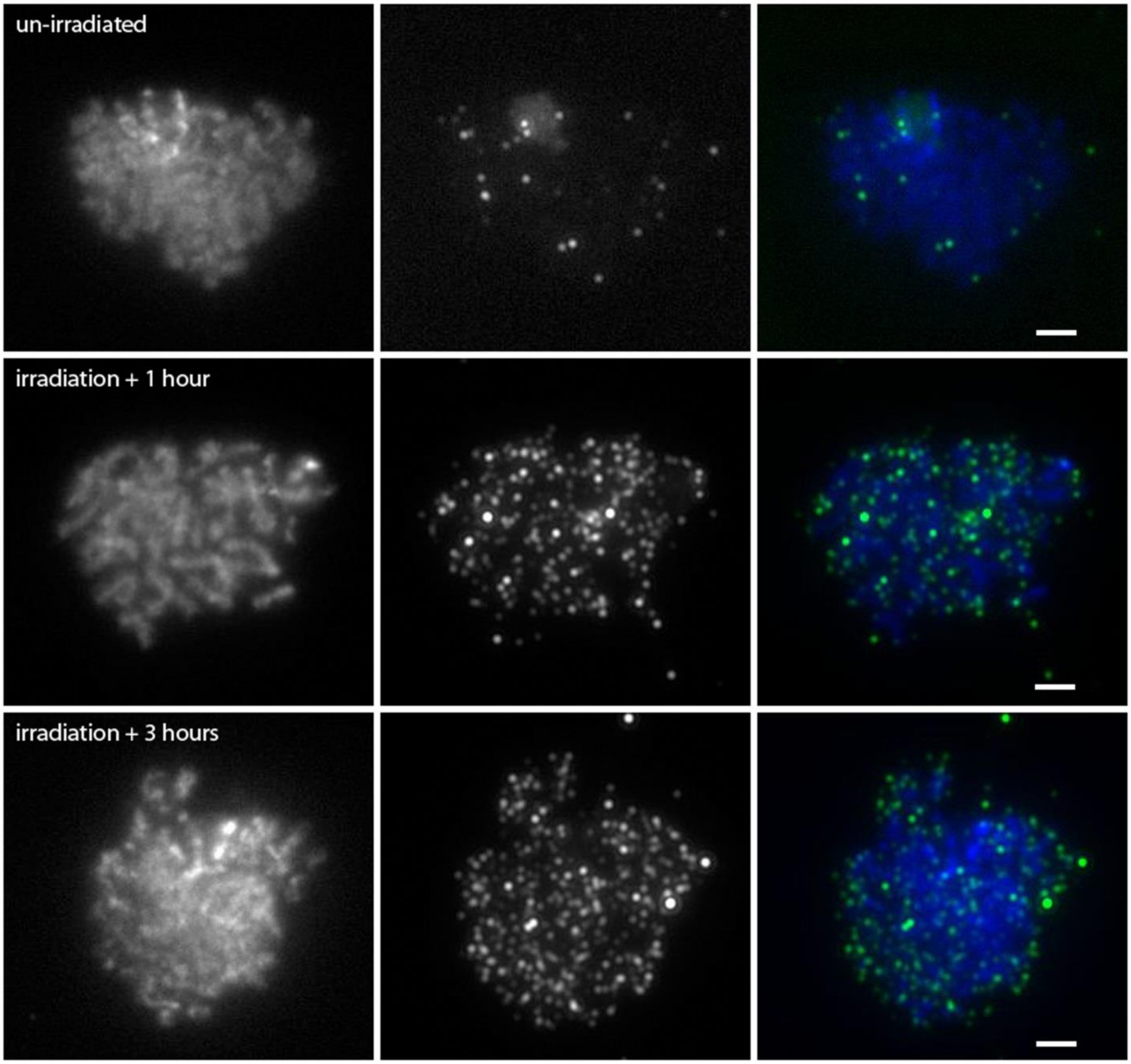

Figure S7 anti-gamma-H2AX localization on *spo11-1* meiotic chromosome spreads from unirradiated mushrooms at 2 hours past karyogamy (n=31), and one hour (n=32) and three hours(n=32) after irradiation with 60 krads. Images in the left hand column are DAPI stained chromosome spreads, images in the middle column are anti-gamma-H2AX counter-stained with a TRITC conjugated secondary antibody and images in the right hand column are the color combined images. Scale bars represent 2 µm.

Table S1 Primer sequences used to amplify *C. cinereus nbs1*

Primer name	Primer sequence
UTR5	TGTTGGGCCCGTTCGACGG
Nbs1Forward56	AAGATGCCCAAATCCTTGTG
Nbs1Forward425	ACGTTTGACCCAGCTAAGGA
Nbs1Forward619	GCCTCCTGTCAGCAAGTACC
Nbs1Forward809	ATTCGGGTGTGACCAAATTC
Nbs1Forward1179	CCCTCCTATCAAGGTGTGGA
Nbs1Forward 1563	CTTTGACGATGGGTTCCATT
Nbs1Forward1773	TGAGACGAACAGGGTTGTGA
Nbs1Forward2100	AGATGATTCGGGGATTAGGG
Nbs1Forward2241	AAAAATGTCCCGAGAAGTGG
Nbs1Forward3159	CAAGGCTGTTGCTTCGATTC
Nbs1Reverse809	GAATTTGGTCACACCCGAAT
Nbs1Reverse1517	GACGAGTCTGCTGGTGGTTC
Nbs1Reverse1843	TCGAGACAGTTGCAGTGGAC
Nbs1Reverse2040	TCGGGTTTGGAAATCTTGAG
Nbs1Reverse2833	TTCTCGCCCTAGTCCTCGTA

Table S2 Primers used to amplify simple sequence repeats on chromosomes 3 and 8

SSR	Forward Primer (with M13 tail)	Reverse Primer
tssr53	TGTAAAACGACGGCCAGTTGCCGGCGTTGTAAGTTTTA	AGAACTCGAATGGTTCAACG
tssr63	TGTAAAACGACGGCCAGTTTCGCTCTTCCAAACAACAA	TGGGACTTTGCAACCTATCC
tssr64	TGTAAAACGACGGCCAGTCTTCCACTTCCGTTTCCTCA	TCCCTATGTCGGATGACGAT
tssr65	TGTAAAACGACGGCCAGTCAATCGTAAACGCAAACGAA	GAGAGGAAGAGGAGGGAGGA
tssr70	TGTAAAACGACGGCCAGTGCGGCCAGACATAACAGAAT	CGATGCCCTTCTGATCTCTT
tssr73	TGTAAAACGACGGCCAGTGAGAAGGTCCACCGGTTTG	GATCACGTGCGGTCAATG
tssr74	TGTAAAACGACGGCCAGTGGAGAGTATCGAGGCGGATG	AGCAATAGCATCGTCAATCG
tssr77	TGTAAAACGACGGCCAGTGCAGCGTCACTCACCACTT	GCAGCTCTCCTGCTCAAACT
tssr78	TGTAAAACGACGGCCAGTCTTCAGTCGCGCAAGTTTC	ATATTGGCTTCGGACAATGC
tssr89	TGTAAAACGACGGCCAGTAAATACCCGGTCCATGATGA	CAATTGGGGAGGGTGTAAAG
tssr93	TGTAAAACGACGGCCAGTTGGGAGGAAGCCATAACTGT	GGGTTGTTGTTTTTGGGTGT
tssr98	TGTAAAACGACGGCCAGTAACAACGACAACACCGTCTG	GCCTAATACCGACGACGACA
tssr108	TGTAAAACGACGGCCAGTGAACGACACCTCCACTCCTC	TGTGTGTTTGTCTCGTCGAA
tssr286	TGTAAAACGACGGCCAGTTACCACCCTGTTGACGTTGA	TGGTACACACCGTTGAAGGA
tssr287	TGTAAAACGACGGCCAGTCTACACCAGTCGAACCGTCA	CATGGTTCACCACACGATTC
tssr292	TGTAAAACGACGGCCAGTTACATCACGGTGGTCTTGGA	ATCGCGACAGCTGTTTATGA
tssr295	TGTAAAACGACGGCCAGTCTCGCTGCCAATACCTCTTC	TGCTTCCCGAACATCTTCTC
tssr298	TGTAAAACGACGGCCAGTCGAGTCTTGGGCGTCATAGT	TGGACTCGGAAACGAGCTTA
tssr500	TGTAAAACGACGGCCAGTGCTTTTGGTGCAGGCTATGA	GGGTTCCTCCCCACTTCTAC
tssr502	TGTAAAACGACGGCCAGTCCTTACCCGATTTCCTAGCC	GACGTGGTAGAAGCAGTGTCC

Table S3 Size of parental alleles used for genotyping.

ssr	*172*	*J6;5-4*	*nbs1-2 (172)*	*nbs1-2;5-3*
53	220.19	221.11	220.37	223.03
63	211.97	214.81	211.87	212.34
65	220.73	214.94	220.77	221.46
70	213.23	217.5	213.66	217.36
73	215.6	218	216.11	218.86
74	216.11	219.42	216.85	214.15
77	229.47	214	229.95	214.63
78	211.69	214	211.73	214.79
89	194.13	218	194.51	218.61
93	207.1	215.09	207.74	215.3
98	208.84	214.69	208.62	214.95
108	200.43	218	200.43	219.24
286	306.84	317.57	307.19	312.52
287	295.54	313.83	296.23	313.89
292	307.81	n/a	307.68	315.24
295	311.27	309.26	312.06	309.11
298	299.68	318.64	299.68	318.67
500	273.96	270.75	273.81	270.38
502	271.22	267.79	271	267.11

Table S4 Primers used to amplify single nucleotide polymorphisms on chromosomes 3 and 8.

SNP name	Forward primer sequence	Reverse Primer sequence
SNP .065M	TGTAAAACGACGGCCAGTAACCTTGCTTGTTTGGGCTA	TGTGTGCTTGAGTTTGGAATG
SNP .1M	GGTATCCGAGGGTTGAGAGG	CACTACCACCAGCACTACCG
SNP .3M	AGAGGCTTACTGACGCTTCG	TAATTCGCTCAAGGCATGTG
SNP .5M	GGACAAAGGACCAGGAATGA	CTTTCGGCTTTCAGTTCGAC
SNP .8M	GGCGAAGAATAAGCGTCAAG	ACCGCAAACTCAACCTATGG
SNP .153	GTCTACACCGGGTCTCTGGA	GAACGCAGTAATCGTGCTCA
SNP .205	CCGTCTCTGAAGAGCCTTTG	ATAACAAGCAGGGCGATGAC
SNP 2.28	TGTAAAACGACGGCCAGTTTGTTCCCCAGAGCTGACTT	ACGTGCCAATGGATGGTAAG

Table S5 Single nucleotide polymorphisms used for genetic mapping

SNP name	location on chromosome	sequence in J6;5-4 and nbs1-2;5-3	sequence in 172 and nbs1-2 (172)
SNP .065M	65309	AGG------TCG	AGGC**ACGAA**TCG
SNP .1M	99606	CC**GCC**TTCG	CC---TTCG
SNP .3M	299500	GTC**C**AGC	GTC**A**AGC
SNP .5M	553545	GAAATT**T**CTGGAGTC	GAAATT-CTGGAGTC
SNP .8M	798676	CATCGTT------TCATTCAC	CATCATT**CCCGCT**TCATTCAC
SNP .153	153710	PCR fragment size 1000 bp	PCR fragment size 1300 bp
SNP .205	205249	GC**C**TGGGTTTATTTATG**G**A**CG**CTAA**C**CG	GC**A**TGGGTTTATTTATG**C**A**GA**CTAA**A**CG
SNP 2.28	2280834	AGA--AC---GGA	AGA**GA**AC**TTG**GGA

Table S6   Interference on chromosome 3

Genetic interval	Wild type	*nbs1-2*
A/B/C	0	0
B/C/D	0	3.33*
C/D/E	0	0
D/E/F	0	2.32
E/F/G	0	2.56
F/G/H	16.23	0
G/H/I	0	0
H/I/J	0	0
I/J/K	0	0
J/K/L	0	0
K/L/M	0	0.67
L/M/N	0	0

*statistically significant, $p < 0.05$

Interference was calculated as in Malkova et al (2004). Zero indicates complete interference, numbers greater than 1 indicate negative interference, and numbers between zero and one indicate loss of positive interference.

Table S7 Interference among chromosome 8 hotspots

Genetic interval	Wild type	*nbs1-2*
A/B/C	1.4	0
B/C/D	0	0.75
H/I/J	0	3.57
