# Supplementary material for: A Mutation in the FHA Domain of Coprinus cinereus Nbs1 Leads to Spo11-Independent Meiotic Recombination and Chromosome Segregation
Source: G3 (Bethesda). 2013 Nov 1;3(11):1927–43. doi: 10.1534/g3.113.007906 (PMC3815056; doi:10.1534/g3.113.007906)
Supplement: Supporting Information [file supp_g3.113.007906_FigureS1.pdf]

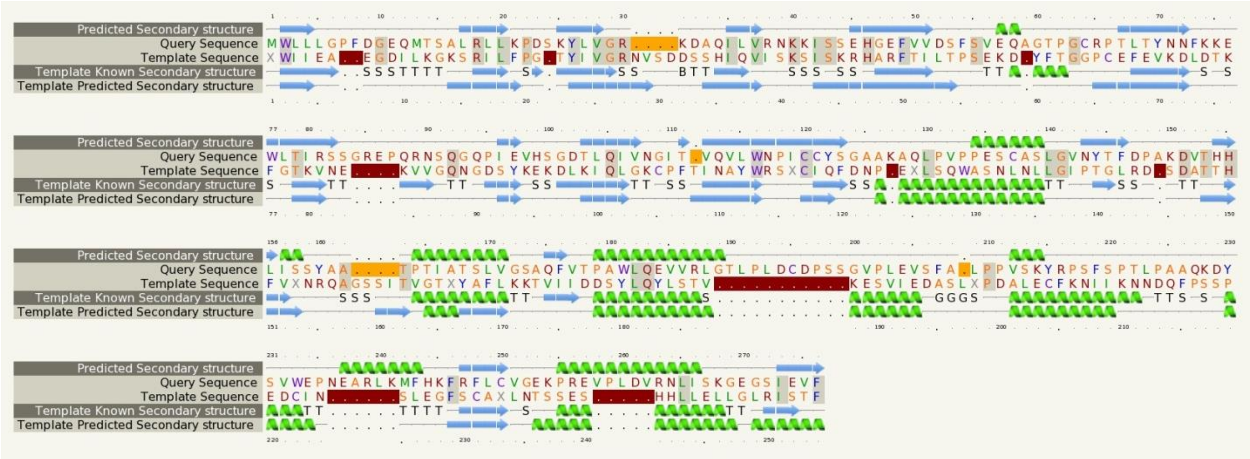

Figure S1 PHYRE2 alignment of *C. cinereus* Nbs1 and *S. pombe* Nbs1 (Kelley and Sternberg 2009). The query sequence is *C. cinereus* Nbs1 and the template sequence is *S. pombe* Nbs1. Predicted secondary structures are made by the PHYRE2 program, and the *S. pombe* known secondary structure is from the crystal structure (Williams et al. 2009). Blue arrows represent beta sheets and green spirals represent alpha helices. Amino acids highlighted in red represent insertions in the *C. cinereus* protein sequence relative to *S. pombe* and those highlighted in yellow represent deletions in the *C. cinereus* protein sequence relative to *S. pombe.* Amino acids 1-115 of *C. cinereus* Nbs1 correspond to the FHA domain of *S. pombe* Nbs1.
