# Supplementary material for: A Mutation in the FHA Domain of Coprinus cinereus Nbs1 Leads to Spo11-Independent Meiotic Recombination and Chromosome Segregation
Source: G3 (Bethesda). 2013 Nov 1;3(11):1927–43. doi: 10.1534/g3.113.007906 (PMC3815056; doi:10.1534/g3.113.007906)
Supplement: Supporting Information [file supp_g3.113.007906_FigureS2.pdf]

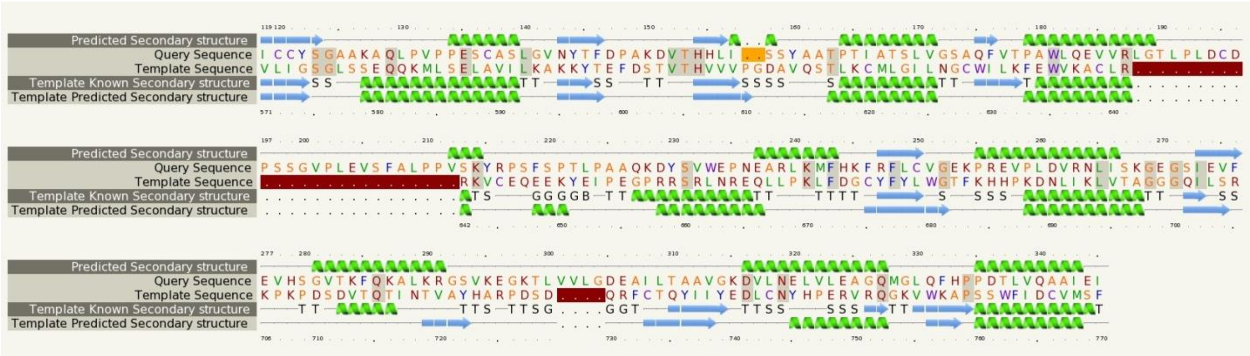

Figure S2 PHYRE2 alignment of *C. cinereus* Nbs1 and human Bard1 BRCT tandem repeat (Kelley and Sternberg 2009). The query sequence is *C. cinereue* Nbs1 and the template sequence is the human Bard1 BRCT tandem repeat . Predicted secondary structures are made by the PHYRE2 program, and human known secondary structure is from the crystal structure. Blue arrows represent beta sheets and green spirals represent alpha helices. Amino acids highlighted in red represent insertions in the *C. cinereus* protein sequence relative to human and those highlighted in yellow represent deletions in the *C. cinereus* protein sequence relative to human. The BRCT1 domain of *C. cinereus* Nbs1 is from amino acids 119-237 and the BRCT2 domain is from amino acids 246-345 of *C. cinereus*.
