# Supplementary material for: A Mutation in the FHA Domain of Coprinus cinereus Nbs1 Leads to Spo11-Independent Meiotic Recombination and Chromosome Segregation
Source: G3 (Bethesda). 2013 Nov 1;3(11):1927–43. doi: 10.1534/g3.113.007906 (PMC3815056; doi:10.1534/g3.113.007906)
Supplement: Supporting Information [file supp_g3.113.007906_FigureS3.pdf]

```
Coprinus    GKKREDEFDREFNRLKISKPDLDAQNGREPEEDWKVLADFGDDSGIRGNFMTICELEVFK  749
            GKKRE + D  ++  +IS  D                    DDS +    + + E
Human       GKKRELKEDSLWSAKEISNND-----------------KLQDDSEMLPKKLLLTEFRSLV  663

Coprinus    EKNGRNAKSAGEMRPEWEGKPNFKKFKRKNVPRSG  784
             KN   +  +G +  ++    NFKKFK+   P +G
Human       IKNSTSRNPSG-INDDYGQLKNFKKFKKVTYPGAG  697
```

Figure S3 Alignment of the Mre11 binding motif (amino acids 770-776) and surrounding sequence from *C. cinereus* and human Nbs1. The *C. cinereus* protein sequence was used as a query in a BLAST against the human protein sequence to produce an alignment.
