# Supplementary material for: A Mutation in the FHA Domain of Coprinus cinereus Nbs1 Leads to Spo11-Independent Meiotic Recombination and Chromosome Segregation
Source: G3 (Bethesda). 2013 Nov 1;3(11):1927–43. doi: 10.1534/g3.113.007906 (PMC3815056; doi:10.1534/g3.113.007906)
Supplement: Supporting Information [file supp_g3.113.007906_FigureS4.pdf]

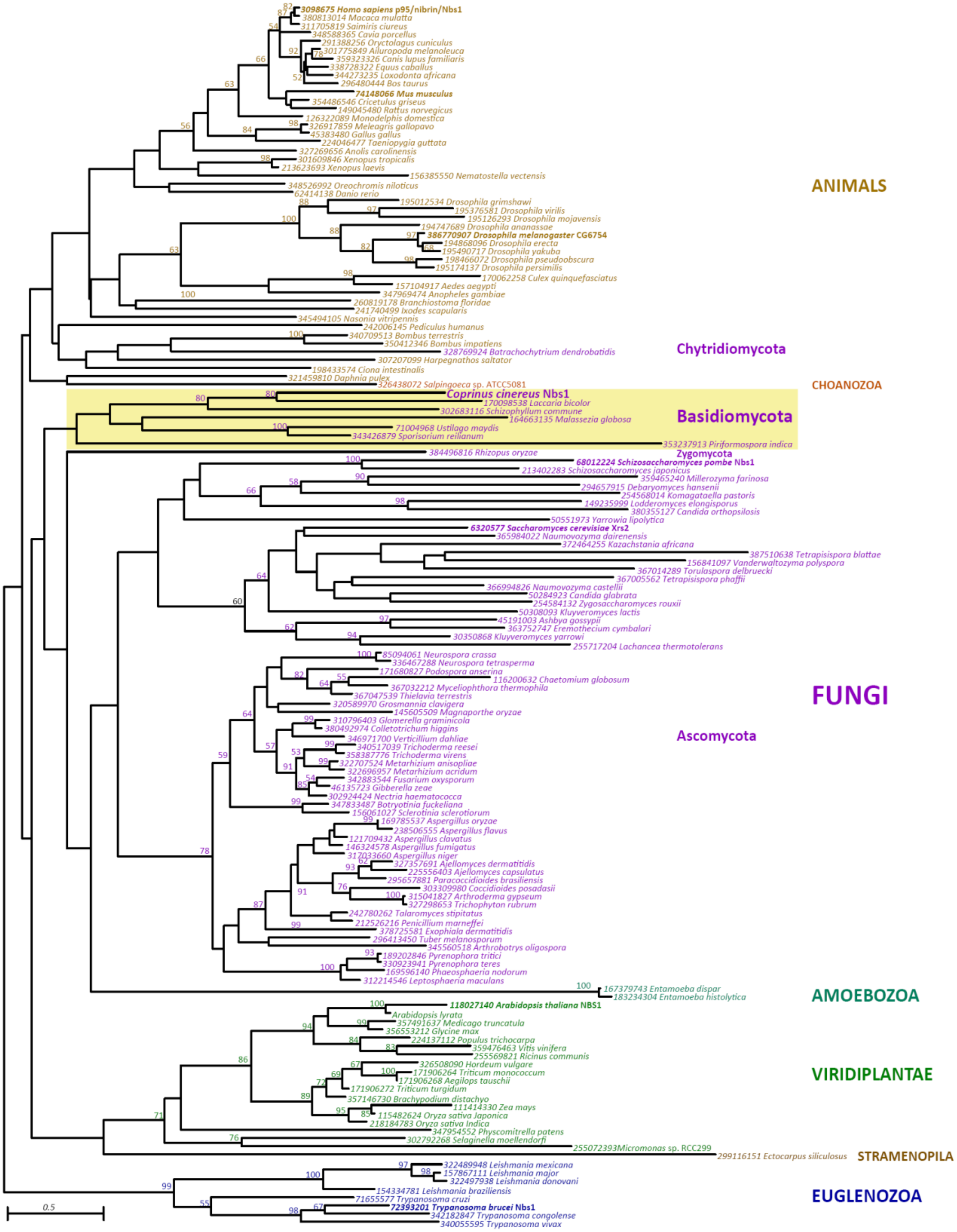
3098675 Homo sapiens p95/nibrin/Nbs1
380813014 Macaca mulatta
311705819 Saimiris ciureus
348588365 Cavia porcellus
291388256 Oryctolagus cuniculus
301775849 Ailuropoda melanoleuca
359323326 Canis lupus familiaris
338728322 Equus caballus
344273235 Loxodonta africana
296480444 Bos taurus
74148066 Mus musculus
354486546 Cricetulus griseus
149045480 Rattus norvegicus
126322089 Monodelphis domestica
326917859 Meleagris gallopavo
45383480 Gallus gallus
224046477 Taeniopygia guttata
327269656 Anolis carolinensis
301609846 Xenopus tropicalis
213623693 Xenopus laevis
156385550 Nematostella vectensis
348526992 Oreochromis niloticus
62414138 Danio rerio
ANIMALS
195012534 Drosophila grimshawi
195376581 Drosophila virilis
195126293 Drosophila mojavensis
194747689 Drosophila ananassae
386770907 Drosophila melanogaster CG6754
194868096 Drosophila erecta
195490717 Drosophila yakuba
198466072 Drosophila pseudoobscura
195174137 Drosophila persimilis
170062258 Culex quinquefasciatus
157104917 Aedes aegypti
347969474 Anopheles gambiae
260819178 Branchiostoma floridae
241740499 Ixodes scapularis
345494105 Nasonia vitripennis
242006145 Pediculus humanus
340709513 Bombus terrestris
350412346 Bombus impatiens
328769924 Batrachochytrium dendrobatidis
Chytridiomycota
307207099 Harpegnathos saltator
198433574 Ciona intestinalis
321459810 Daphnia pulex
326438072 Salpingoeca sp. ATCC5081
CHOANOZOA
Coprinus cinereus Nbs1
170098538 Laccaria bicolor
302683116 Schizophyllum commune
164663135 Malassezia globosa
Basidiomycota
71004968 Ustilago maydis
343426879 Sporisorium reilianum
353237913 Piriformospora indica
384496816 Rhizopus oryzae
Zygomycota
68012224 Schizosaccharomyces pombe Nbs1
213402283 Schizosaccharomyces japonicus
359465240 Millerozyma farinosa
294657915 Debaryomyces hansenii
254568014 Komagataella pastoris
149235999 Lodderomyces elongisporus
380355127 Candida orthopsilosis
50551973 Yarrowia lipolytica
6320577 Saccharomyces cerevisiae Xrs2
365984022 Naumovozyma dairenensis
372464255 Kazachstania africana
387510638 Tetrapisispora blattae
156841097 Vanderwaltozyma polyspora
367014289 Torulaspora delbrueckii
367005562 Tetrapisispora phaffii
366994826 Naumovozyma castellii
50284923 Candida glabrata
254584132 Zygosaccharomyces rouxii
50308093 Kluyveromyces lactis
45191003 Ashbya gossypii
363752747 Eremothecium cymbalari
30350868 Kluyveromyces yarrowi
255717204 Lachancea thermotolerans
85094061 Neurospora crassa
336467288 Neurospora tetrasperma
171680827 Podospora anserina
116200632 Chaetomium globosum
367032212 Myceliophthora thermophila
367047539 Thielavia terrestris
320589970 Grosmannia clavigera
145605509 Magnaporthe oryzae
FUNGI
310796403 Glomerella graminicola
380492974 Colletotrichum higgins
Ascomycota
346971700 Verticillium dahliae
340517039 Trichoderma reesei
358387776 Trichoderma virens
322707524 Metarhizium anisopliae
322696957 Metarhizium acridum
342883544 Fusarium oxysporum
46135723 Gibberella zeae
302924424 Nectria haematococca
347833487 Botryotinia fuckeliana
156061027 Sclerotinia sclerotiorum
169785537 Aspergillus oryzae
238506555 Aspergillus flavus
121709432 Aspergillus clavatus
146324578 Aspergillus fumigatus
317033660 Aspergillus niger
327357691 Ajellomyces dermatitidis
225556403 Ajellomyces capsulatus
295657881 Paracoccidioides brasiliensis
303309980 Coccidioides posadasii
315041827 Arthroderma gypseum
327298653 Trichophyton rubrum
242780262 Talaromyces stipitatus
212526216 Penicillium marneffei
378725581 Exophiala dermatitidis
296413450 Tuber melanosporum
345560518 Arthrobotrys oligospora
189202846 Pyrenophora tritici
330923941 Pyrenophora teres
169596140 Phaeosphaeria nodorum
312214546 Leptosphaeria maculans
167379743 Entamoeba dispar
183234304 Entamoeba histolytica
AMOEBOZOA
118027140 Arabidopsis thaliana NBS1
Arabidopsis lyrata
357491637 Medicago truncatula
356553212 Glycine max
224137112 Populus trichocarpa
359476463 Vitis vinifera
255569821 Ricinus communis
326508090 Hordeum vulgare
171906264 Triticum monococcum
171906268 Aegilops tauschii
171906272 Triticum turgidum
357146730 Brachypodium distachyo
111414330 Zea mays
115482624 Oryza sativa Japonica
218184783 Oryza sativa Indica
347954552 Physcomitrella patens
302792268 Selaginella moellendorfi
255072393Micromonas sp. RCC299
VIRIDIPLANTAE
299116151 Ectocarpus siliculosus
STRAMENOPILA
322489948 Leishmania mexicana
157867111 Leishmania major
322497938 Leishmania donovani
154334781 Leishmania braziliensis
71655577 Trypanosoma cruzi
72393201 Trypanosoma brucei Nbs1
342182847 Trypanosoma congolense
340055595 Trypanosoma vivax
EUGLENOZOA
0.5

Figure S4 Phylogenetic tree showing the evolutionary relationships among eukaryotic orthologs of *nbs1* from animals, fungi, plants and protists. *C. cinereus* Nbs1 groups with its closest relative *Laccaria* and other Basidiomycete fungi. Branch support is indicated by numbers at the base of branches, the percent boostrap support $\geq$ 50%. The scale bar represents the distance of 0.5 amino acid substitutions per site. 186 aligned amino acid sites were analyzed using the LG+25γ substitution model implemented in RAxML version 7.3.1, resulting in this tree with an optimized LnL=-41564.91. The parameter describing the γ-distributed amino acid substitution frequencies is α=1.58. The GenInfo Identifier numbers for each sequence from Genbank are indicated.
