# Supplementary material for: A Mutation in the FHA Domain of Coprinus cinereus Nbs1 Leads to Spo11-Independent Meiotic Recombination and Chromosome Segregation
Source: G3 (Bethesda). 2013 Nov 1;3(11):1927–43. doi: 10.1534/g3.113.007906 (PMC3815056; doi:10.1534/g3.113.007906)
Supplement: Supporting Information [file supp_g3.113.007906_FigureS5.pdf]

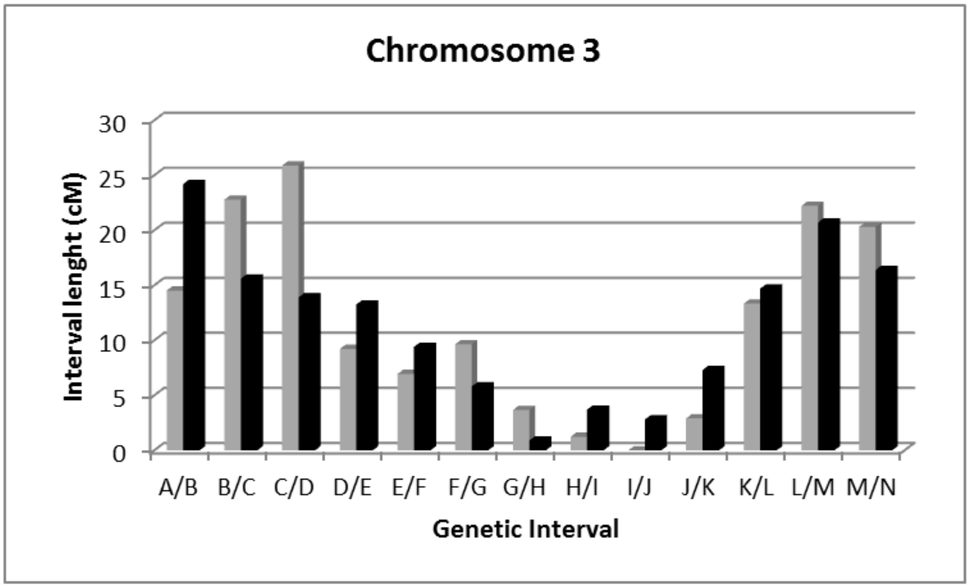

Figure S5 Map lengths of individual intervals along chromosome 3 from the homozygous and heterozygous nbs1-2 crosses. Grey bars are the homokaryon nbs1-2 cross and black bars are the heterokaryon wild type x nbs1-2 cross. No interval is statistically significantly different, nor is the total map lengths.
