# Supplementary material for: A Mutation in the FHA Domain of Coprinus cinereus Nbs1 Leads to Spo11-Independent Meiotic Recombination and Chromosome Segregation
Source: G3 (Bethesda). 2013 Nov 1;3(11):1927–43. doi: 10.1534/g3.113.007906 (PMC3815056; doi:10.1534/g3.113.007906)
Supplement: Supporting Information [file supp_g3.113.007906_FigureS6.pdf]

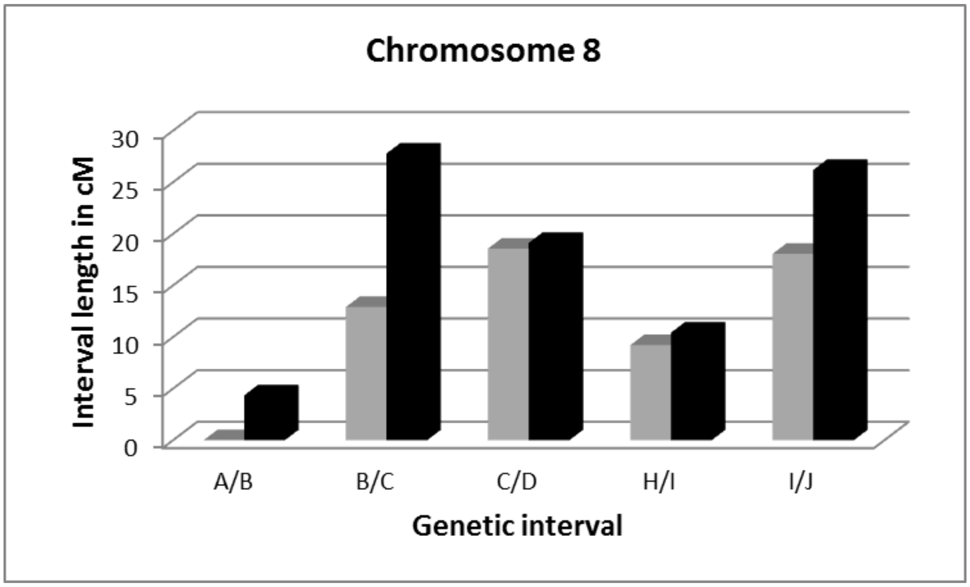

Figure S6 Map lengths of individual hotspot intervals on chromosome 8. Light grey bars are the homokaryon nbs1-2 cross and black bars are the heterokaryon wild type x nbs1-2 cross. Total map lengths are not statistically significantly different.
