# Supplementary material for: A Mutation in the FHA Domain of Coprinus cinereus Nbs1 Leads to Spo11-Independent Meiotic Recombination and Chromosome Segregation
Source: G3 (Bethesda). 2013 Nov 1;3(11):1927–43. doi: 10.1534/g3.113.007906 (PMC3815056; doi:10.1534/g3.113.007906)
Supplement: Supporting Information [file supp_g3.113.007906_FigureS7.pdf]

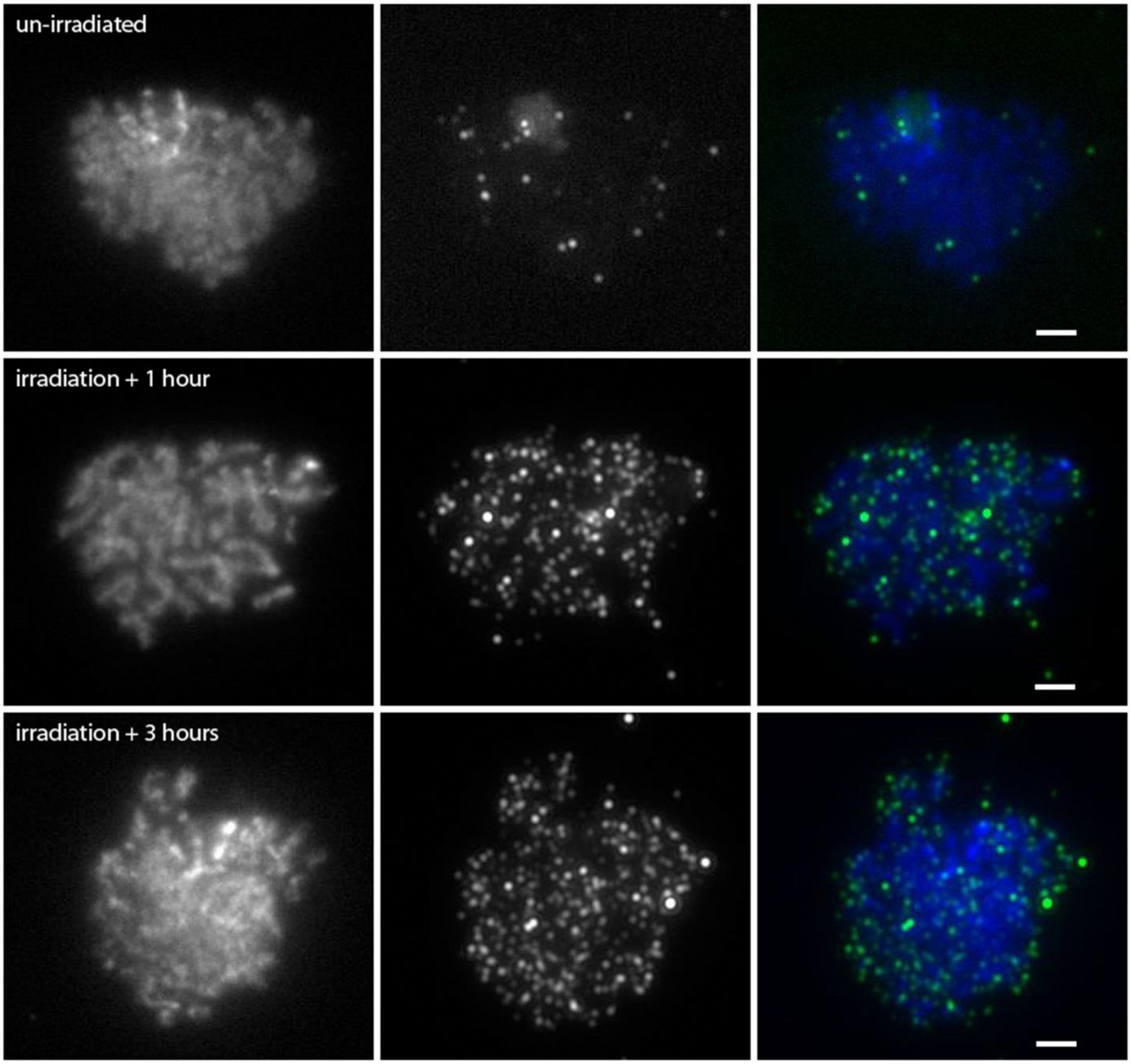

Figure S7 anti-gamma-H2AX localization on *spo11-1* meiotic chromosome spreads from unirradiated mushrooms at 2 hours past karyogamy (n=31), and one hour (n=32) and three hours(n=32) after irradiation with 60 krads. Images in the left hand column are DAPI stained chromosome spreads, images in the middle column are anti-gamma-H2AX counter-stained with a TRITC conjugated secondary antibody and images in the right hand column are the color combined images. Scale bars represent 2 µm.
