# Supplementary material for: A Mutation in the FHA Domain of Coprinus cinereus Nbs1 Leads to Spo11-Independent Meiotic Recombination and Chromosome Segregation
Source: G3 (Bethesda). 2013 Nov 1;3(11):1927–43. doi: 10.1534/g3.113.007906 (PMC3815056; doi:10.1534/g3.113.007906)
Supplement: Supporting Information [file supp_g3.113.007906_TableS1.pdf]

Table S1 Primer sequences used to amplify *C. cinereus nbs1*

Primer name	Primer sequence
UTR5	TGTTGGGCCCGTTCGACGG
Nbs1Forward56	AAGATGCCCAAATCCTTGTG
Nbs1Forward425	ACGTTTGACCCAGCTAAGGA
Nbs1Forward619	GCCTCCTGTCAGCAAGTACC
Nbs1Forward809	ATTCGGGTGTGACCAAATTC
Nbs1Forward1179	CCCTCCTATCAAGGTGTGGA
Nbs1Forward 1563	CTTTGACGATGGGTTCCATT
Nbs1Forward1773	TGAGACGAACAGGGTTGTGA
Nbs1Forward2100	AGATGATTCGGGGATTAGGG
Nbs1Forward2241	AAAAATGTCCCGAGAAGTGG
Nbs1Forward3159	CAAGGCTGTTGCTTCGATTC
Nbs1Reverse809	GAATTTGGTCACACCCGAAT
Nbs1Reverse1517	GACGAGTCTGCTGGTGGTTC
Nbs1Reverse1843	TCGAGACAGTTGCAGTGGAC
Nbs1Reverse2040	TCGGGTTTGGAAATCTTGAG
Nbs1Reverse2833	TTCTCGCCCTAGTCCTCGTA
