# Supplementary material for: A Mutation in the FHA Domain of Coprinus cinereus Nbs1 Leads to Spo11-Independent Meiotic Recombination and Chromosome Segregation
Source: G3 (Bethesda). 2013 Nov 1;3(11):1927–43. doi: 10.1534/g3.113.007906 (PMC3815056; doi:10.1534/g3.113.007906)
Supplement: Supporting Information [file supp_g3.113.007906_TableS2.pdf]

Table S2 Primers used to amplify simple sequence repeats on chromosomes 3 and 8

SSR	Forward Primer (with M13 tail)	Reverse Primer
tssr53	TGTAAAACGACGGCCAGTTGCCGGCGTTGTAAGTTTTA	AGAACTCGAATGGTTCAACG
tssr63	TGTAAAACGACGGCCAGTTTCGCTCTTCCAAACAACAA	TGGGACTTTGCAACCTATCC
tssr64	TGTAAAACGACGGCCAGTCTTCCACTTCCGTTTCCTCA	TCCCTATGTCGGATGACGAT
tssr65	TGTAAAACGACGGCCAGTCAATCGTAAACGCAAACGAA	GAGAGGAAGAGGAGGGAGGA
tssr70	TGTAAAACGACGGCCAGTGCGGCCAGACATAACAGAAT	CGATGCCCTTCTGATCTCTT
tssr73	TGTAAAACGACGGCCAGTGAGAAGGTCCACCGGTTTG	GATCACGTGCGGTCAATG
tssr74	TGTAAAACGACGGCCAGTGGAGAGTATCGAGGCGGATG	AGCAATAGCATCGTCAATCG
tssr77	TGTAAAACGACGGCCAGTGCAGCGTCACTCACCACTT	GCAGCTCTCCTGCTCAAACT
tssr78	TGTAAAACGACGGCCAGTCTTCAGTCGCGCAAGTTTC	ATATTGGCTTCGGACAATGC
tssr89	TGTAAAACGACGGCCAGTAAATACCCGGTCCATGATGA	CAATTGGGGAGGGTGTAAAG
tssr93	TGTAAAACGACGGCCAGTTGGGAGGAAGCCATAACTGT	GGGTTGTTGTTTTTGGGTGT
tssr98	TGTAAAACGACGGCCAGTAACAACGACAACACCGTCTG	GCCTAATACCGACGACGACA
tssr108	TGTAAAACGACGGCCAGTGAACGACACCTCCACTCCTC	TGTGTGTTTGTCTCGTCGAA
tssr286	TGTAAAACGACGGCCAGTTACCACCCTGTTGACGTTGA	TGGTACACACCGTTGAAGGA
tssr287	TGTAAAACGACGGCCAGTCTACACCAGTCGAACCGTCA	CATGGTTCACCACACGATTC
tssr292	TGTAAAACGACGGCCAGTTACATCACGGTGGTCTTGGA	ATCGCGACAGCTGTTTATGA
tssr295	TGTAAAACGACGGCCAGTCTCGCTGCCAATACCTCTTC	TGCTTCCCGAACATCTTCTC
tssr298	TGTAAAACGACGGCCAGTCGAGTCTTGGGCGTCATAGT	TGGACTCGGAAACGAGCTTA
tssr500	TGTAAAACGACGGCCAGTGCTTTTGGTGCAGGCTATGA	GGGTTCCTCCCCACTTCTAC
tssr502	TGTAAAACGACGGCCAGTCCTTACCCGATTTCCTAGCC	GACGTGGTAGAAGCAGTGTCC
