# Supplementary material for: A Mutation in the FHA Domain of Coprinus cinereus Nbs1 Leads to Spo11-Independent Meiotic Recombination and Chromosome Segregation
Source: G3 (Bethesda). 2013 Nov 1;3(11):1927–43. doi: 10.1534/g3.113.007906 (PMC3815056; doi:10.1534/g3.113.007906)
Supplement: Supporting Information [file supp_g3.113.007906_TableS3.pdf]

Table S3 Size of parental alleles used for genotyping.

ssr	*172*	*J6;5-4*	*nbs1-2 (172)*	*nbs1-2;5-3*
53	220.19	221.11	220.37	223.03
63	211.97	214.81	211.87	212.34
65	220.73	214.94	220.77	221.46
70	213.23	217.5	213.66	217.36
73	215.6	218	216.11	218.86
74	216.11	219.42	216.85	214.15
77	229.47	214	229.95	214.63
78	211.69	214	211.73	214.79
89	194.13	218	194.51	218.61
93	207.1	215.09	207.74	215.3
98	208.84	214.69	208.62	214.95
108	200.43	218	200.43	219.24
286	306.84	317.57	307.19	312.52
287	295.54	313.83	296.23	313.89
292	307.81	n/a	307.68	315.24
295	311.27	309.26	312.06	309.11
298	299.68	318.64	299.68	318.67
500	273.96	270.75	273.81	270.38
502	271.22	267.79	271	267.11
