# Supplementary material for: A Mutation in the FHA Domain of Coprinus cinereus Nbs1 Leads to Spo11-Independent Meiotic Recombination and Chromosome Segregation
Source: G3 (Bethesda). 2013 Nov 1;3(11):1927–43. doi: 10.1534/g3.113.007906 (PMC3815056; doi:10.1534/g3.113.007906)
Supplement: Supporting Information [file supp_g3.113.007906_TableS4.pdf]

Table S4 Primers used to amplify single nucleotide polymorphisms on chromosomes 3 and 8.

SNP name	Forward primer sequence	Reverse Primer sequence
SNP .065M	TGTAAAACGACGGCCAGTAACCTTGCTTGTTTGGGCTA	TGTGTGCTTGAGTTTGGAATG
SNP .1M	GGTATCCGAGGGTTGAGAGG	CACTACCACCAGCACTACCG
SNP .3M	AGAGGCTTACTGACGCTTCG	TAATTCGCTCAAGGCATGTG
SNP .5M	GGACAAAGGACCAGGAATGA	CTTTCGGCTTTCAGTTCGAC
SNP .8M	GGCGAAGAATAAGCGTCAAG	ACCGCAAACTCAACCTATGG
SNP .153	GTCTACACCGGGTCTCTGGA	GAACGCAGTAATCGTGCTCA
SNP .205	CCGTCTCTGAAGAGCCTTTG	ATAACAAGCAGGGCGATGAC
SNP 2.28	TGTAAAACGACGGCCAGTTTGTTCCCCAGAGCTGACTT	ACGTGCCAATGGATGGTAAG
