# Supplementary material for: A Mutation in the FHA Domain of Coprinus cinereus Nbs1 Leads to Spo11-Independent Meiotic Recombination and Chromosome Segregation
Source: G3 (Bethesda). 2013 Nov 1;3(11):1927–43. doi: 10.1534/g3.113.007906 (PMC3815056; doi:10.1534/g3.113.007906)
Supplement: Supporting Information [file supp_g3.113.007906_TableS5.pdf]

Table S5 Single nucleotide polymorphisms used for genetic mapping

SNP name	location on chromosome	sequence in J6;5-4 and nbs1-2;5-3	sequence in 172 and nbs1-2 (172)
SNP .065M	65309	AGG------TCG	AGGC**ACGAA**TCG
SNP .1M	99606	CC**GCC**TTCG	CC---TTCG
SNP .3M	299500	GTC**C**AGC	GTC**A**AGC
SNP .5M	553545	GAAATT**T**CTGGAGTC	GAAATT-CTGGAGTC
SNP .8M	798676	CATCGTT------TCATTCAC	CATCATT**CCCGCT**TCATTCAC
SNP .153	153710	PCR fragment size 1000 bp	PCR fragment size 1300 bp
SNP .205	205249	GC**C**TGGGTTTATTTATG**G**A**CG**CTAA**C**CG	GC**A**TGGGTTTATTTATG**C**A**GA**CTAA**A**CG
SNP 2.28	2280834	AGA--AC---GGA	AGA**GA**AC**TTG**GGA
