# Supplementary material for: A Mutation in the FHA Domain of Coprinus cinereus Nbs1 Leads to Spo11-Independent Meiotic Recombination and Chromosome Segregation
Source: G3 (Bethesda). 2013 Nov 1;3(11):1927–43. doi: 10.1534/g3.113.007906 (PMC3815056; doi:10.1534/g3.113.007906)
Supplement: Supporting Information [file supp_g3.113.007906_TableS6.pdf]

Table S6   Interference on chromosome 3

Genetic interval	Wild type	*nbs1-2*
A/B/C	0	0
B/C/D	0	3.33*
C/D/E	0	0
D/E/F	0	2.32
E/F/G	0	2.56
F/G/H	16.23	0
G/H/I	0	0
H/I/J	0	0
I/J/K	0	0
J/K/L	0	0
K/L/M	0	0.67
L/M/N	0	0

*statistically significant, $p < 0.05$

Interference was calculated as in Malkova et al (2004). Zero indicates complete interference, numbers greater than 1 indicate negative interference, and numbers between zero and one indicate loss of positive interference.
