# Supplementary material for: A Mutation in the FHA Domain of Coprinus cinereus Nbs1 Leads to Spo11-Independent Meiotic Recombination and Chromosome Segregation
Source: G3 (Bethesda). 2013 Nov 1;3(11):1927–43. doi: 10.1534/g3.113.007906 (PMC3815056; doi:10.1534/g3.113.007906)
Supplement: Supporting Information [file supp_g3.113.007906_TableS7.pdf]

Table S7 Interference among chromosome 8 hotspots

Genetic interval	Wild type	*nbs1-2*
A/B/C	1.4	0
B/C/D	0	0.75
H/I/J	0	3.57
